# Arabidopsis Novel Microgametophyte Defective Mutant 1 Is Required for Pollen Viability *via* Influencing Intine Development in Arabidopsis

**DOI:** 10.3389/fpls.2022.814870

**Published:** 2022-04-12

**Authors:** Limin Mi, Aowei Mo, Jiange Yang, Hui Liu, Ding Ren, Wanli Chen, Haifei Long, Ning Jiang, Tian Zhang, Pingli Lu

**Affiliations:** ^1^School of Life Sciences, Fudan University, Shanghai, China; ^2^State Key Laboratory of Crop Stress Adaptation and Improvement, School of Life Sciences, Henan University, Kaifeng, China

**Keywords:** male fertility, pollen viability, pollen wall, intine, AtNMDM1

## Abstract

The pollen intine layer is necessary for male fertility in flowering plants. However, the mechanisms behind the developmental regulation of intine formation still remain largely unknown. Here, we identified a positive regulator, *Arabidopsis novel microgametophyte defective mutant 1* (*AtNMDM1*), which influences male fertility by regulating intine formation. The *AtNMDM1*, encoding a pollen nuclei-localized protein, was highly expressed in the pollens at the late anther stages, 10–12. Both the mutations and the knock-down of *AtNMDM1* resulted in pollen defects and significantly lowered the seed-setting rates. Genetic transmission analysis indicated that *AtNMDM1* is a microgametophyte lethal gene. Calcofluor white staining revealed that abnormal cellulose distribution was present in the aborted pollen. Ultrastructural analyses showed that the abnormal intine rather than the exine led to pollen abortion. We further found, using transcriptome analysis, that cell wall modification was the most highly enriched gene ontology (GO) term used in the category of biological processes. Notably, two categories of genes, *Arabinogalactan proteins* (*AGPs*) and *pectin methylesterases* (*PMEs*) were greatly reduced, which were associated with pollen intine formation. In addition, we also identified another regulator, AtNMDM2, which interacted with AtNMDM1 in the pollen nuclei. Taken together, we identified a novel regulator, AtNMDM1 that affected cellulose distribution in the intine by regulating intine-related gene expression; furthermore, these results provide insights into the molecular mechanisms of pollen intine development.

## Introduction

In higher plants, flowers are the organs of sexual reproduction. In *Arabidopsis thaliana*, a mature flower is generally composed of four parts: sepals, petals, stamens, and pistils (Ma, 2005). Stamens have two parts; one is short and the other is long. According to morphological observation, the development of Arabidopsis flowers can be divided into 20 stages. This starts with floral primordia at flower stages, 1 and 2. At flower stage 3, sepal primordia are present. The floral bud is gradually enveloped by the sepals until flower stage 6. At the same time, petal and stamen primordia are beginning to appear at flower stage 5 and the gynoecium becomes visible at flower stage 6. At flower stage 7, the long stamen primordia sticks to the base. The shape of petal primordia start to be similar to ellipse. At flower stage 8, locules begin to form in the long stamens. Petals are still at the base of the floral bud and the size of the organ continues to increase at flower stage 9. The rapidly growing petals reach the top of the short stamens at flower stage 10. Stigmatic papillae are clearly visible at flower stage 11 and petals grow to the same height as the long stamens at flower stage 12. Following anthesis at flower stage 13, fertilization takes place at flower stage 14. Siliques continue growing until the seeds mature and fall at between stages 15 and 20 (Smyth et al., 1990; Alvarez-Buylla et al., 2010).

Anther development begins at flowering stage 5, in which stamen primordia start to form. Based on the morphological analysis, anther development can be divided into 14 stages. At anther stages, 1–5, the stamen primordia go through division and differentiation and gradually form various tissue types of anthers until the pattern of the anther is defined. Microspore mother cells appear at anther stage 5. These processes of anther development take place at flower stages 5–9. From anther stages 6–9, after the completion of meiosis in the microspore mother cells, the microspores are subsequently released from the tetrad and undergo vacuolization and the middle layer of the anther walls starts to degenerate. At the end of anther stage 9, the middle layer is almost degraded, the size of the tapetum in the anther wall gradually increases, and the cells of the tapetum become vacuolated. These processes occur at flower stage 10. At anther stage 10, the tapetum begins to degenerate and the microspores enter the first asymmetric mitosis. At anther stage 11, microspores enter the second mitosis. The endothecium layer thickens significantly, and fibrous bands are deposited in the endothecium layer and the connective tissue. The septum cells between the two compartments begin to degenerate and stomium cells begin to differentiate. At anther stage 12, tricellular pollen, including one vegetative cell and two sperm cells, appear in the anther. Anther stages 10–12 correspond to flower stages 11 and 12. At the anther stage 13, stomium cells degenerate and mature pollen is released from the locules. These developmental processes occur at flower stages 13 and 14 (Sanders et al., 1999; Alvarez-Buylla et al., 2010).

In flowering plants, the pollen wall is important for the success of sexual reproduction as it both protects the microgametophyte from various biotic and abiotic stresses, and also functions in cell–cell recognition during pollination (Zinkl et al., 1999; Edlund et al., 2004). In Arabidopsis, the mature pollen wall has an intricate multi-layered structure, including the intine, exine, and tryphine (Owen and Makaroff, 1995; Yue et al., 2014; Shi et al., 2015). The exine is comprised of two layers: an outer sculpted sexine layer and an inner nexine layer (Ariizumi and Toriyama, 2011; Yue et al., 2014). The double-layered structure, present next to the pollen plasma membrane, is called the intine and is composed of exintine and endintine (Ariizumi and Toriyama, 2011). The pattern of the exine is initially established at the tetrad stage (the layer is called primexine), while that of the intine takes place at the developmental stage of microspore, which is later than the initiation of the exine (Owen and Makaroff, 1995; Li et al., 2010; Jiang et al., 2013). In rice, the intine of pollen begins to form at the binuclear pollen stage (Zhang and Wilson, 2009; Li and Zhang, 2010). Exine formation is thought to be regulated by sporophytes, whereas the development of the intine is mainly determined by the male gametophyte (Van Aelst et al., 1993; Shi et al., 2015).

The pollen exine is primarily made up of a tough material called sporopollenin, which is composed of fatty acid derivatives and phenylpropanoids (Morant et al., 2007). Genetic studies have revealed a number of genes involved in the formation of pollen exine in Arabidopsis, such as *defective in exine formation 1* (*DEX1*), *no exine formation 1 (NEF1)*, *ruptured pollen grain 2 (RPG2)*, *no primexine and plasma membrane undulation (NPU)*, *exine formation defect (EFD)*, *acyl-CoA synthetase 5 (ACOS5)*, *CYP703A2, CYP704B1*, and *callose defective microspore 1 (CDM1)* (Paxson-Sowders et al., 2001; Morant et al., 2007; Dobritsa et al., 2009; Souza et al., 2009; Chang et al., 2012; Sun et al., 2013; Hu et al., 2014; Lu et al., 2014). Functional studies have revealed that the mutation of these genes can affect the normal formation of exine by influencing primexine formation and/or sporopollenin synthesis, consequently leading to reduced pollen viability.

Generally, it is thought that the pollen intine is composed of pectin, cellulose, hemicellulose, hydrolytic enzymes, and arabinogalactan proteins (Knox, 1971; Knox and Heslop-Harrison, 1971; Aelst and Went, 1992; Hasegawa et al., 2000; Li et al., 2010; Shi et al., 2015). Till date, only a few genes have been shown to be individually involved in pollen intine formation. Arabidopsis uridine diphosphate sugar pyrophosphorylase (AtUSP) disrupts the formation of the pectocellulosic intine without affecting the exine (Schnurr et al., 2006). *CESA3*, the cellulose synthase (CESA) gene, encodes the catalytic subunits of the cellulose synthase complexes. However, the intine is hardly visible following staining with Calcofluor white in *CESA3* mutants (Persson et al., 2007). The fasciclin-like arabinogalactan protein 3 (FLA3), which is tightly anchored to the plasma membrane, greatly affects the formation of pollen intine (Li et al., 2010). Furthermore, in rice, three genes, *rice immature pollen 1 (RIP1*), *glycosyltransferase 1* (*OsGT1*), and *collapsed abnormal pollen 1* (*CAP1*), are required for intine development (Han et al., 2006; Moon et al., 2013; Ueda et al., 2013). More recent studies have shown that a microRNA OsmiR528 directly acts on chimeric AGP OsUCL23 to regulate the transport and accumulation of various metabolites, especially flavonoids, thereby regulating the development of the pollen intine layer (Zhang et al., 2020). In *Brassica campestris*, silencing of *pectate lyase-like* (*PLL*) genes, such as *BcPLL9* or *BcPLL10*, causes abnormal intine development (Jiang et al., 2014a). From the above-mentioned studies, it is clear that genes involved in cell wall synthesis or modification appear to be crucial for pollen intine development.

The molecular mechanism controlling intine development remains largely unknown, potentially owing to difficulties in observing the defects in the pollen intine of the relevant mutants. Here, we report a microgametophyte lethal gene, *AtNMDM1* encoding a putative transcription coactivator that is located in pollen nuclei. We reveal that dysfunction of the novel regulator, AtNMDM1 downregulates the intine-related gene expression, leading to abnormal cellulose distribution in the intine thereby leading to pollen defect. Our findings provide novel insights into the role of a regulator in the mechanism of intine development.

## Materials and Methods

### Plant Materials and Growth Conditions

The wild type used in this study was Col-0. The *AtNMDM1*^+ / −^ mutants were generated by the CRISPR/Cas9 system (Wang et al., 2015; Xie et al., 2015). The seeds were surface sterilized and grown on plates with 1/2 Murashige and Skoog (MS) medium salt. After growth on agar for 2 weeks, the seedlings were transferred into a greenhouse. The seeds were also sown in the soil directly. All Arabidopsis plants were grown under long-day (16-h light/8-h dark) and ambient light conditions in a greenhouse at 22°C.

### Mutant Acquisition and Genotype

The CRISPR/Cas9 genome editing system was used to generate genome editing mutants of *AtNMDM1* (Wang et al., 2015; Xie et al., 2015). The specific spacer sequences were selected using the CRISPR-PLANT database^1^ and the construction was generated as previously described (Xie et al., 2015). The construct was confirmed by sequencing and then transformed into the wild type using Agrobacterium-mediated techniques with the floral dip method. The positive independent T1 transgenic plants having resistance for hygromycin were selected. To identify whether a gene was edited, direct-sequencing was performed. In detail, genomic DNA was isolated from young inflorescences of transgenic plants to amplify the fragments containing the designed targeted sites and by sequencing the PCR products. To obtain the mutated variants of genes, complex sequencing was carried out. We performed PCR amplification for individuals, subsequently cloning the mixed PCR products into the T vectors for Sanger sequencing. The frequency of a specific *Atnmdm1* allele was calculated as the ratio between the number of T clones with this particular mutation and the total number of T clones with all the different genotypes were detected.

### RNA Extraction and Reverse Transcription-Quantitative Real-Time PCR

To estimate the RNA expression level, the total RNA of samples was extracted using TRIzol Reagent (Invitrogen, United States; 15596018) according to the manufacturer’s instructions. A FastQuant RT kit was used to synthesize the first-strand complementary DNA (cDNA) as described above (TIANGEN, China; KR106). A sample of cDNA was subjected to reverse transcription-quantitative real-time PCR (RT-qPCR) in the final volume of 20 μL by using a SuperReal PreMix Plus (TIANGEN; FP205) with an ABI StepOnePlus real-time PCR System (Life Technologies, United States). The RT-qPCR primers were designed by NCBI Primer-BLAST^2^ using the qPrimerDB-qPCR Primer Database^3^. The data were normalized to the expression levels of the internal control genes, *ACT2* (*AT3G18780*) or *EF2*α (*AT5G60390*).

### β-Glucuronidase Staining

The β-glucuronidase (GUS) reporter construct was generated by introducing the promotor of *AtNMDM1* (1546 bp before “ATG”) into a pCambia1381 plasmid with two restriction sites, *Pst*I and *Nco*I using the two primers, *Pst*I-pT3GUS-F and *Nco*I-pT3GUS-R. The plasmid was transformed into GV3101, the *Agrobacterium tumefaciens* strain, and introduced into Col-0 wild-type plants using *Agrobacterium*-mediated techniques and the floral dip method (Clough and Bent, 1998). Positive plants were screened on a hygromycin selection plate. The selection plate contained a half-strength MS medium with 0.6% agar. Independent transgenic plants underwent GUS staining. Various tissues of *ProAtNMDM1:GUS* transgenic plants were treated with 90% of acetone on ice for 10 min. Then the samples were washed several times with GUS staining buffer [10 μM of ethylenediaminetetraacetic acid (EDTA) (pH 8.0), 50 mM of Na_2_HPO_4_, 50 mM of NaH_2_PO_4_, 0.5 mM of K_4_Fe(CN)_6_, 0.5 mM of K_3_Fe(CN)_6_, 0.1% of Triton X-100, and 20% of methanol]. Subsequently, the GUS staining buffer was removed. The materials were vacuum infiltrated with the GUS mix buffer [GUS staining buffer and 0.1% of 5-bromo-4-chloro-3-indolyl-β-D-glucuronide (X-Gluc) in N, N-dimethylformamide] for a few minutes and then incubated at 37°C overnight. For colored samples, the chlorophyll was removed using 100% of ethanol.

### RNAi Assay

*LAT52* promoter is specifically expressed in the pollen (Twell et al., 1990; Eady et al., 1994). In this study, wild-type (Col-0) plants were transformed with a pMeio-LAT52 construct harboring a hairpin *AtNMDM1*-RNAi cassette (Zhong et al., 2012). Two pairs of primers, AtNMDM1_Ri_*Nco*I and AtNMDM1_Ri_*ApaI*, were used to amplify the sense fragment, and AtNMDM1-Ri-*Xba*I and AtNMDM1-Ri-*Bst*EII were used to amplify the antisense fragment. The plasmid was transformed into *Agrobacterium* (GV3101) and introduced into Col-0 wild-type plants using *Agrobacterium*-mediated techniques with the floral dip method. Positive plants were screened in a hygromycin-resistant plate. Hygromycin-resistant *AtNMDM1*-RNAi transgenic plants were selected for further study. To eliminate the transgenic effects during the T1 generation, we performed further examination of T3 plants and verified the expression level of *AtNMDM1* at the anther stages 9–13 by RT-qPCR.

### Phenotypic Analyses

Plant parts (siliques and flowers) were photographed with a Canon digital camera on a Nikon dissecting microscope. Inflorescences were collected and fixed in Carnoy’s fixative (75% of alcohol, 25% of glacial acetic acid). Pollen grains were stained with Alexander red solution following a published protocol (Peterson et al., 2010). Tetrads were stained with 0.01% of toluidine blue. For callose examination, tetrads were dissected from the anthers and stained with 0.01% (w/v) of aniline blue as described previously (Lu et al., 2014). Semi-thin sections of anthers from the flowers of mutants, RNAi lines, and the wild type were fixed overnight in FAA (50% of ethanol, 5.0% of glacial acid, and 3.7% of formaldehyde), dehydrated in a graded ethanol series (2 × 50, 70, 85, 95, 2 × 100%), and then embedded in Technovit 7100 resin (Heraeus Kulzer) as previously described (Zhang et al., 2007; Wang et al., 2017). The embedded tissues were sectioned (1μm thick) using a motorized RM2265 rotary microtome (Leica Microsystems). The sections were stained with 0.05% of toluidine blue and photographed under an AXIO ScopeA1 microscope. Different anther stages were observed according to the previous description of morphological landmarks in the anther (Sanders et al., 1999).

To observe the pollen intine, sections and pollen grains were stained with a mix of Calcofluor white stain and 10% of potassium hydroxide at a 1:1 ratio (v/v) for 5 min (0.5 mg/mL; Sigma-Aldrich Cat No 18909). Subsequently, the stained sections were observed and photographed under an AXIO ScopeA1 microscope with a UV light. Autofluorescence of sexine is an indication of exine (Lou et al., 2014). For TEM observation, different stages of flower buds were fixed in 2.5% of glutaric dialdehyde buffer and vacuumed for 1 h, then embedded into the fresh mixed resin and polymerized in molds, sectioned, and observed under a TEM microscopy (JEOL, Japan) as previously described (Lou et al., 2014).

### Confocal Laser Scanning Microscope for Green Fluorescent Protein Imaging

Subcellular localization of AtNMDM1 infused with an enhanced green fluorescent protein (eGFP) was carried out on the pollen of transgenic plants with the *ProAtNMDM1:AtNMDM1-eGFP* construct. We used the homologous recombination method to introduce the promoter of *AtNMDM1* (1546 bp upstream before ATG) into a pCambia1302-eGFP binary vector with two primers, pT3-pCambia1302EB-F and pT3-pCambia1302EB-R. Then, two other primers, cds1_*Pst*I_F and cds1_*Nco*I-R, were used to amplify *AtNMDM1* CDS. The fragments were inserted into the *ProAtNMDM1:eGFP* binary vector with two restriction sites, *Pst*I and *Nco*I. For developmental analysis, the pollen from buds at different stages of development was teased out of the anther with a needle and mounted directly in DAPI solution. GFP signals in the transformed pollen were photographed under a confocal laser scanning microscope (Leica TCS SP8).

### Sequence Alignment

Gene annotation was found on the Arabidopsis Information Resource^4^. Domain prediction was performed using the NCBI conserved domain tool^5^. Protein sequences of homologs to *AtNMDM1* were identified using BLASTP of the NCBI^6^. Multiple sequence alignments were generated by MEGA v.6.0 using the neighbor-joining method and default values^7^.

### RNA-seq and Gene Ontology Enrichment Analysis

Anthers at stages 8 and 9 were selected according to the morphology of anthers and flower buds, as previously described (Smyth et al., 1990; Sanders et al., 1999). More than 250 anthers at stages 8 and 9 from the wild type and the mutant plants were harvested under a dissecting microscope and immediately frozen in liquid nitrogen. Total RNA from each sample was extracted and purified using the TRIzol Reagent (Invitrogen; 15596018). At least 2 μg of total RNA per sample were used for deep sequencing with an Illumina Hisequation 2000 system. The raw sequence data were collected and filtered. Approximately, 19 million reads from the wild type and the mutants were obtained for nuclear-encoded genes. The significance of differentially expressed genes (DGEs) were determined using *P* < 0.05 and | log2 (FoldChange)| > 1. The GO was used for the categorization of significant DEGs between the wild type and *Atnmdm1-5/*+ to identify those genes involved in the distinct biological processes, including the biological process, molecular function, and cellular component. The GO and enrichment analysis of the functional categories were performed with the AgriGO toolkit (Tian et al., 2017). The raw sequencing data have been submitted to the NCBI BioProject database^8^ under accession number PRJNA797722.

### Protein Extraction, Western Blot, and Immunoprecipitation-Mass Spectrometry Data Analysis

Total proteins were extracted in a lysis buffer (7 M of urea, 2 M of thiourea, 30 mM of tris) from 3 to 5 inflorescences. Protein samples were collected by centrifugation at 12,000 *g* for 15 min at 4°C. Total proteins were separated by SDS-PAGE, transferred to a nitrocellulose membrane, immunoblotted with different antibodies, and detected using the Clarity Western ECL Substrate (BIORad). Antibody Flag tag (mouse) (Cat^#^GNI 4110-FG, Shanghai genomics technology) was used as the primary antibody, and goat anti-mouse HRP-conjugated secondary antibody (Cat^#^GNI 9310-M, Shanghai Genomics Technology) was used for fluorescence detection. Signals were visualized with a CLiNX Science Instruments imager. Immunoprecipitation (IP) of proteins interacting with AtNMDM1-3 × FLAG infused protein from an inflorescence was performed and the IP samples were analyzed by an immunoprecipitation mass spectrometry (IP/MS). FLAG-tagged proteins were recognized and bound by the anti-FLAG M2 antibody and specifically pulled-down by M2-conjugated agarose beads. Mass spectrometry data were selected by two criteria. One was that the bait proteins were only obtained in *ProAtNMDM1:AtNMDM1-FLAG* transgenic plant; the second was that the value of peptide spectrum matches (PSMs) were greater than or equal to 3 compared to the wild type. The PSMs displayed the total number of identified peptide sequences for the protein, including those redundantly identified. The GO analysis of functional category analyses was performed by AgriGO (Tian et al., 2017). Subsequently, non-redundant and highly significant GO terms were performed and visualized using the web tool REVIGO (Supek et al., 2011).

### Bimolecular Fluorescence Complementation Analysis

The full lengths of *AtNMDM1* and *AtNMDM1* CDS in Arabidopsis were cloned into pXY104/pXY106 using *Bam*HI*/Sal*I sites. Then plasmids were transformed into *Agrobacterium* GV3101 cells. Transformants were harvested once the OD_600_ reached 2.0, and were resuspended in MES/MgCl_2_/AS solution to a final OD_600_ of 1.0. Cell suspensions were mixed at a 1:1 ratio of various combinations, and young *Nicotiana benthamiana* leaves were infiltrated. Leaves were excised and visualized under a confocal microscope (Leica TCS SP8) following an incubation period of 48 h.

### Primer Sequences

Primers used in this study are listed in the Supplementary Data Set 7.

### Accession Numbers

Protein sequence data used in sequence alignment were download from NCBI under the accession numbers listed in Figure 6. Homolog sequences were shown as the following accession numbers: NP_196487 (*Arabidopsis thaliana*); XP_003602657 (*Medicago truncatula*); XP_002309936 (*Populus trichocarpa*); XP_019234270 (*Nicotiana attenuata*); XP_015623703 (*Oryza sativa*); XP_003575132 (*Brachypodium distachyon*); ACG39505 (*Zea mays*); XP_024367313 (*Physcomitrella patens*); AAT72821.1 (*Human sapiens*); NP_035424 (*Mus musculus*); NP_001009618 (*Rattus norvegicus*); NP_501750 (*Caenorhabditis elegans*); NP_477136 (*Drosophila melanogaster*); AJS89849 (*Saccharomyces cerevisiae*); *AtNMDM1* (AT5G09250, *Arabidopsis thaliana*); and *AtNMDM2* (AT4G10920, *Arabidopsis thaliana*).

## Results

### The Identification of a Microgametophyte Defective Gene in Arabidopsis

To deeply understand the genetic and molecular mechanism controlling the microgametophyte development in flowering plants, it is necessary to uncover critical genes involved in this process. In mammals, a study using testis tissue revealed more than 50 chromatin-associated proteins involved in meiotic processes (Luo et al., 2013). From the evolutionary conservation of many genes in the reproductive processes, it is possible that the homologs of the above-mentioned mouse genes in Arabidopsis might also function in the microgametophyte development of plants. Therefore, we searched the homologs in Arabidopsis for those proteins and identified 10 candidate genes for further study (Supplementary Data Set 1). Subsequently, we employed the clustered regularly interspaced short palindromic repeats (CRISPR)/Cas9-based genome-editing approach to generate the corresponding mutations for further functional studies. In the same vector, two targeted sites were designed separately for two candidate genes. Among the obtained genome-editing mutants, we found that five T1 plants showed severe fertility defects, in which *At5g09250* and *At1g77250* were the target sites. Direct-sequencing results indicated that site 1 of the gene, *At5g09250* rather than the gene, *At1g77250* was edited in the five T1 lines (Supplementary Figure 1). Thus, we named the responsible gene (*At5g09250*) as *Arabidopsis novel microgametophyte defective mutant 1* (*AtNMDM1*).

**FIGURE 1:**
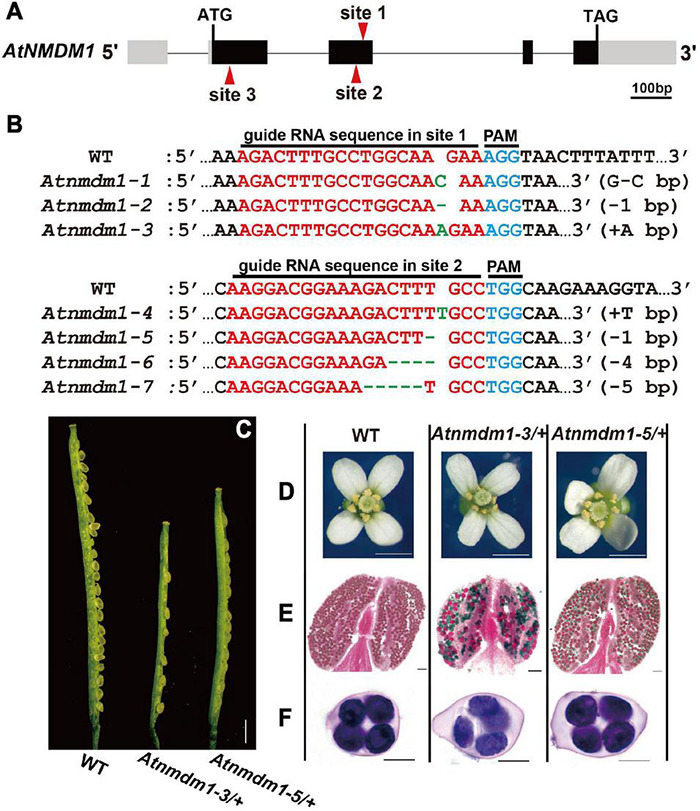
Genotype and phenotype analysis of *AtNMDM1*^+ / −^ mutants. **(A)** The genomic structure of the *AtNMDM1* from Arabidopsis. Exons are represented by black boxes. Introns are shown by a dashed line. The three target sites (site 1, site 2, and site 3) are indicated by red arrows. The length of *AtNMDM1* is 1344 bp. **(B)** The various mutations of the *AtNMDM1* allele were generated by the CRISPR/Cas9 genome-editing system in T1 plants. The wild-type sequence is shown at the top with the PAM sequence highlighted in blue and the target sequence in red. Green dashes and letters indicate deleted bases and insertions or changed bases, separately (+, insertion; –, deletion; letter-letter, changed). **(C–F)** The phenotypes of the *AtNMDM1*^+ / −^ mutants, *Atnmdm1-3/*+ and *Atnmdm1-5/*+ were analyzed. **(C)** Silique length of *Atnmdm1-3/*+ and *Atnmdm1-5/*+ were obviously shorter than in the wild type (WT) and unfertilized ovules were obvious in *Atnmdm1-3/*+ and *Atnmdm1-5/*+. Bar = 1 cm. **(D)** The number of pollen grains in *Atnmdm1-3/*+ and *Atnmdm1-5/*+ were less than in the wild type. Bar = 1 mm. **(E)** Alexander staining of anthers of wild type and *Atnmdm1-3/*+ and *Atnmdm1-5/*+. Note that normal pollen grains are stained red while dead pollens are stained green. *Atnmdm1-3/*+ and *Atnmdm1-5/*+ both displayed around half of their pollen being dead. Bar = 50 μm. **(F)** No difference was observed when tetrads were stained with toluidine blue in the wild type and *AtNMDM1*^+ / −^ mutants: *Atnmdm1-3/*+ and *Atnmdm1-5/*+. Bar = 10 μm.

To obtain a detailed genotype of *AtNMDM1*, we randomly selected three T1 plants with representative fertility defects for sequencing the targeted sites using the complex-sequencing method. Sequencing results revealed that the wild-type allele was detected in the above three lines. Three types of mutations, namely, G-to-C base editing (G-C), a deletion of one base pair (−1 bp), and an insertion of A (+A bp) at site 1 of *AtNMDM1*, are shown in Figure 1B. These results indicated the presence of three heterozygous lines of *AtNMDM1*. We named the three mutated alleles as *Atnmdm1-1* (G-C), *Atnmdm1-2* (−1 bp), and *Atnmdm1-3* (+A bp) (Figure 1B).

In conclusion, we identified the novel gene, *AtNMDM1* involved in the development of microgametophytes.

### Generation of Additional *AtNMDM1* Mutant Alleles

Based on the information available in the Arabidopsis Information Resource (TAIR), the *AtNMDM1* gene is thought to be 1,344 bp long, containing four exons and four introns (Figure 1A). To further confirm the function of *AtNMDM1* genetically, we designed two new guide RNA (gRNA) recognition sites (site 2 and site 3) specific to the *AtNMDM1* to generate additional mutants with a CRISPR/Cas9-based genome-editing system (Figure 1A). No *Atnmdm1* mutant was identified, whose gRNA was designed to be present at site 3 of *AtNMDM1* (Figure 1A). We speculated that the AtNMDM1 protein was damaged too severely following the death of the transgenic plant.

At the targeted site 2 of *AtNMDM1*, we gained 45 independent T1 transgenic plants which are resistant to hygromycin (Figure 1A). Among them, we found more than 20 plants having obvious abnormal pollens in the T1 generation. Subsequently, we randomly selected three transgenic plants for further study. We detected four mutated variants at site 2 of *AtNMDM1*, namely, an insertion of T (+T bp), a deletion of one base pair (−1 bp), a deletion of four base pairs (−4 bp), and a deletion of five base pairs (−5 bp) (Figure 1B). Sequencing results also showed that the wild-type allele existed in the above lines. These results strongly implied that all the above lines were heterozygous. Furthermore, we gained four genome-edited alleles at the targeted site 2 in *AtNMDM1*, namely *Atnmdm1-4* (+T bp), *Atnmdm1-5* (−1 bp), *Atnmdm1-6* (−4 bp), and *Atnmdm1-7* (−5 bp) (Figure 1B).

In summary, the analysis of additional *AtNMDM1* mutants further confirmed the function of *AtNMDM1* in microgametophyte development.

### Genotype Analyses of *AtNMDM1* Mutants

To obtain genetically stable homozygous *Atnmdm1* without *Cas9/gRNA* DNA, we screened the T2 population of the selected lines. After genotyping 160 T2 individuals with abnormal pollen phenotypes, we found that these plants possessed the transgenic *Cas9/gRNA* fragment. Subsequently, we analyzed the genotype of more than 10 T2 individuals with pollen defects. Sequencing results indicated that the wild-type allele was detected in the above T2 plants. Two new mutated variants of *AtNMDM1* were detected in the T2 generation: −2 bp at site 1 (*Atnmdm1-8*) and −6 bp at site 2 (*Atnmdm1-9*) (Supplementary Figures 2A,C). New genome-edited alleles in *AtNMDM1* were continually produced in the T2 generation owing to the presence of the Cas9-based editing system.

To confirm the main mutated variant of *AtNMDM1*, we analyzed the frequency of *Atnmdm1* alleles in the two selected T2 plants from two targeted sites, respectively. In the T2 plants edited at site 1 of *AtNMDM1*, we detected a frequency of approximately 50% for *Atnmdm1-3* (Supplementary Figure 2B), 12% for *Atnmdm1-2*, and 38% for the wild type allele (Supplementary Figure 2B). In T2 plants edited at site 2 of *AtNMDM1*, we detected a frequency of approximately 47% for *Atnmdm1-5* allele (Supplementary Figure 2D), 7% for *Atnmdm1-4* allele, 13% for *Atnmdm1-6* allele, 7% for *Atnmdm1-9* allele, and 26% for the wild type allele (Supplementary Figure 2D). Combining this information with the previous sequence analysis of T1 plants (Figure 1B), we concluded that we obtained two heterozygous *AtNMDM1* mutants with pollen defects: *Atnmdm1-3/*+ and *Atnmdm1-5/*+.

### Genetic Analysis and Comparative Phenotypic Analysis of *AtNMDM1*^+ / −^ Mutants

The phenotype of microgametophyte defects were observed in the progeny when *Atnmdm1-3/*+ and *Atnmdm1-5/*+ plants were self-pollinated. The percent of plants with pollen defects in the progeny was around 50% (Supplementary Table 1). The result implied that the genetic transmission of the *Atnmdm1-3* and *Atnmdm1-5* alleles were delivered by gametophytes. To test this hypothesis, we performed reciprocal crosses between *Atnmdm1-5/*+ and the wild type (Supplementary Table 1). After pollinating the stigma of *Atnmdm1-5/*+ plants with the wild-type pollen, we found a frequency of approximately 45% for the transmission of the abnormal pollen phenotype through the female gametophyte, suggesting that the *AtNMDM1* is a microgametophyte lethal gene (Supplementary Table 1). Unexpectedly, when the wild type was crossed with *Atnmdm1-5/*+ plants as the male parent, their progeny yielded a similar transmission frequency of the abnormal pollen phenotype (∼47%) as above (Supplementary Table 1). This result is not consistent with the Mendelian theory, in which the phenotype of all F2 plants should be similar to the wild type. This might be owing to the *Cas9/gRNA* DNA remaining in the F2 plants with an abnormal pollen phenotype. Considering that *AtNMDM1* is a microgametophyte lethal gene and the genetic transmission of *Atnmdm1-3* and *Atnmdm1-5* is delivered by the female parent, genetically stable homozygous lines with microgametophyte defects cannot be obtained.

Next, further phenotypic analyses were performed on the *AtNMDM1*^+ / −^ mutants: *Atnmdm1-3/*+, and *Atnmdm1-5/*+. The vegetative growth of *AtNMDM1*^+ / −^ mutants was quite normal: the number of rosette leaves, cauline leaves, and the height of plants were similar to those of the wild type (Supplementary Figure 2E). For the reproductive stage, *AtNMDM1*^+ / −^, the mutants had similar sepals, petals, and tetrads to the wild type (Figures 1D,F). Compared to the wild type, shorter siliques were produced, and many unfertilized ovules were observed in *AtNMDM1*^+ / −^ mutants (Figure 1C). In *Atnmdm1-5/*+, the number of seeds per silique (26.09 ± 3.97, *n* = 637, n represents the number of siliques counted) was reduced approximately 50% compared to 54 seeds per silique (54.98 ± 4.07, *n* = 306) in the wild type (Supplementary Table 2). Consistent with the reduced fertility, fewer pollen grains were observed on the stigmas of the flowers in the *AtNMDM1*^+ / −^ mutant plants (Figure 1D). Alexander’s staining showed that a significant portion of pollen grains in the anther of *AtNMDM1*^+ / −^ mutants were stained green, indicating they were aborted. In contrast, viable pollen grains in wild-type anthers were stained red (Figure 1E). In summary, the loss-of-function of *AtNMDM1* had no effect on the vegetative growth of the plant, but led to microgametophyte defects after meiosis and significantly reduced the seed-setting ratio.

To further examine the viability of pollen in *Atnmdm1-5/*+ plants, we noted that the average number of seeds per silique can reach 45 (45.61 ± 4.09, *n* = 87) when wild-type stigmas were pollinated with the pollen of *Atnmdm1-5/*+ plants (Supplementary Table 2). This data suggested that most normal-type pollen are viable in *Atnmdm1-5/*+ plants. When pollinating stigmas of *Atnmdm1-5/*+ plants with wild-type pollen, only 25 seeds per silique (25.71 ± 4.22, *n* = 203) were produced, much less than in the wild type (54 seeds per silique) (Supplementary Table 2). This indicated that the development of the female gametophyte was also affected in the *Atnmdm1-5/*+ plant. Therefore, we concluded that *AtNMDM1* was not only involved in microgametophyte development but also in the reproductive development of female gametophytes.

### Temporal and Spatial Expression of *AtNMDM1*

**FIGURE 2:**
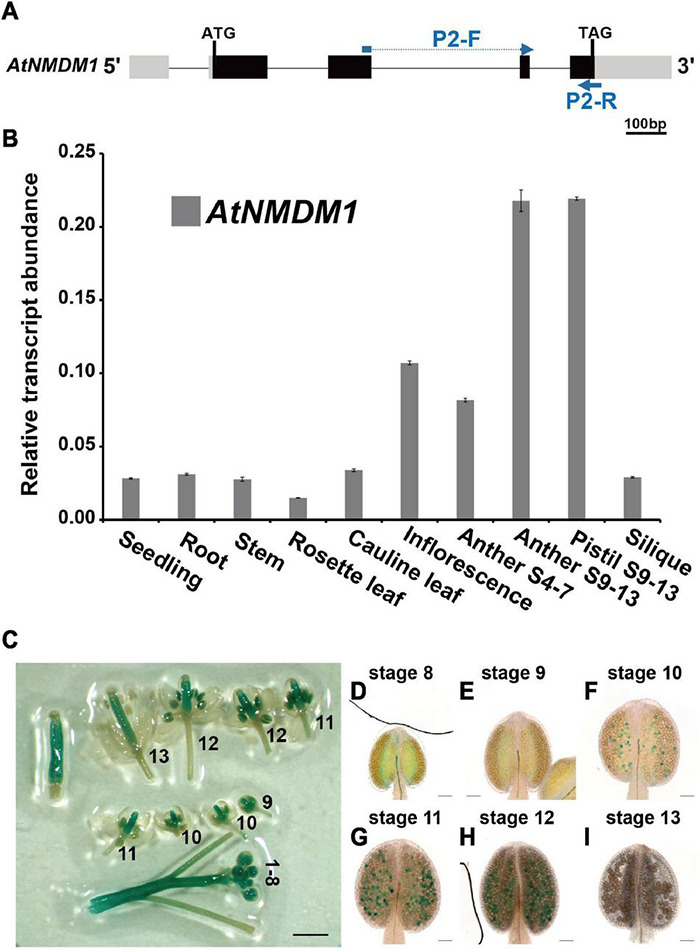
Expression profile analysis of *AtNMDM1* in various tissues. **(A)** The transcript abundance of *AtNMDM1* among different Arabidopsis tissues was determined by real-time PCR and normalized against *ACTIN-2*. The primers, P2-F and P2-R of cDNA fragments in the *AtNMDM1* are indicated by blue arrows. **(B)** The transcription levels of *AtNMDM1* were highly expressed in the anther and pistil at anther stages 9–13 (anther S9–13 and pistil S9–13), compared with the other tissues, including the seedlings, roots, stems, rosette leaves, cauline leaves, inflorescence, anthers stages 4–7 (anther S4–7), and siliques. The bars represent mean ± standard error (SE) of three biological replicates. **(C–I)** *AtNMDM1* promoter triggered *GUS* expression in flowers and anthers at different developmental stages. **(C)** The number represents the developmental stage of the flower. **(D–I)** Anthers are shown at anther stages 8–13. The blue points in the anthers represent the GUS signals in the pollen. bar = 1 mm; **(D–I)**, bar = 50 μm.

To determine the temporal and spatial expression of *AtNMDM1*, RT-qPCR analysis was performed (Figure 2A). We found that *AtNMDM1* was highly expressed in young inflorescence while it was less detectable in the seedlings, roots, stems, rosette leaves, cauline leaves, and siliques (Figure 2B). As anther development in Arabidopsis is divided into 14 distinct stages based on the morphological landmarks (Sanders et al., 1999), we further investigated the *AtNMDM1* transcript level at different developmental stages of the anther and pistil. Our results revealed that the *AtNMDM1* transcript reached its highest expression level in the anther and pistil at anther stages 9–13 (Figure 2B). The abundance of *AtNMDM1* transcript was relatively low during anther stages 4–7 (Figure 2B). Consistent with the RT-qPCR analysis of *AtNMDM1* transcript level, the expression of GUS reporter gene under the *AtNMDM1* promoter was intense in the inflorescence (Figure 2C). The GUS signals were mainly detected in the anthers and pistils at flower stages 1–12. However, at flower stage 13, the GUS signals were weaker in the anther than in the pistil (Figure 2C). We investigated the detailed patterns of *AtNMDM1* expression in the anther during stages 8–13 (Figures 2D,I). Weak GUS signals were detectable in the pollen at anther stages, 8 and 9 (Figures 2D,E), with increasing intensity up to stage 12 (Figures 2F–H). No GUS signal was detectable in the pollen at stage 13 (Figure 2I). In addition, GUS signals were also detected in the seedlings at the different stages and in various tissues and organs, such as stem, young siliques, and ovaries (Supplementary Figures 3A–F and Figure 2C), while low or no signals were detected in the rosette leaves and old siliques (Supplementary Figures 3C,F). Hence, *AtNMDM1* was widely expressed in various tissues of plants, but the highest expression was found in the pistil and stamen of the inflorescence. This is consistent with the function of *AtNMDM1* in the reproductive development of microgametophytes and female gametophytes.

### Male Fertility Reduction in *AtNMDM1* RNAi Transgenic Plants

**FIGURE 3:**
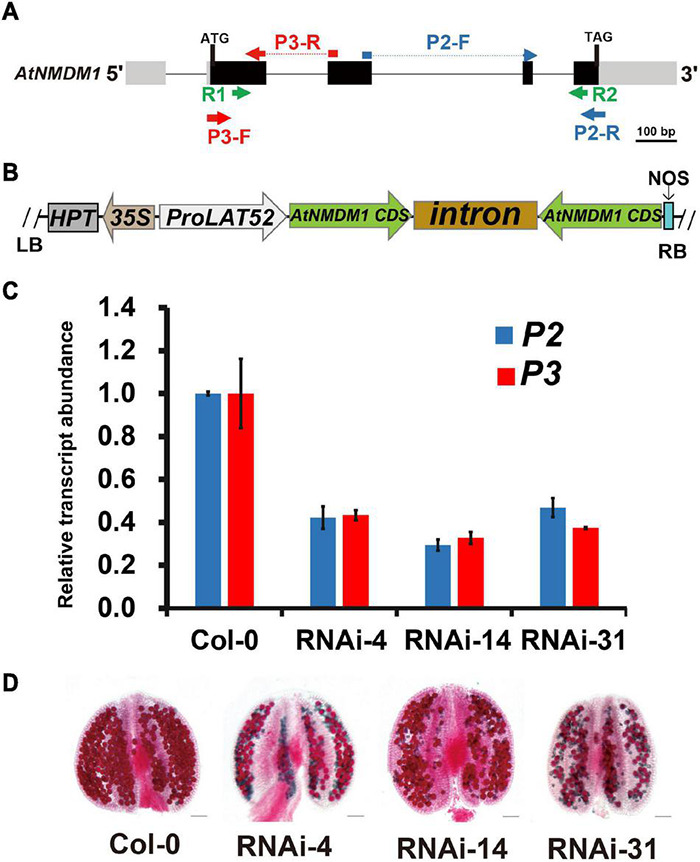
Knockdown of *AtNMDM1* expression in Arabidopsis pollen resulted in defective fertility. **(A)** The genomic structure of *AtNMDM1* from Arabidopsis. Exons are represented by black boxes with the size in base pairs of each exon indicated. The primers, R1/R2 of *AtNMDM1* RNAi fragments are indicated by green arrows. The primers, P3-F/R (red arrow) and P2-F/R (blue arrow) of two different cDNA fragments in *AtNMDM1* are indicated, respectively. **(B)** The *ProLAT52:AtNMDM1-*RNAi construct is represented, which is driven by the Tomato pollen-specific promoter, *LAT52*. RB, right border; LB, left border; HPT, hygromycin phosphotransferase gene; NOS, nopaline synthase terminator. **(C)** The transcript levels of *AtNMDM1* cDNA fragments are significantly reduced in the anther of three *AtNMDM1* RNAi lines RNAi-4, RNAi-14, and RNAi-31. P2 and P3 are two *AtNMDM1* cDNA fragments that are designed for the detection of *AtNMDM1* expression levels. The location information of P2 and P3 is indicated in **(A)**. Mean result from three biological replicates are shown. Data were normalized to *EF1*α expression. The error bar is the mean ± SE of triplicate experiments. **(D)** Alexander staining of anthers in *AtNMDM1* RNAi lines RNAi-4, RNAi-14, and RNAi-31. Bar = 50 μm.

Our results revealed that *AtNMDM1* was involved in the development of both male and female gametophytes, and was highly expressed in the pollen at the later stage (Figure 1, Supplementary Table 2, and Supplementary Figure 2). To validate the function of *AtNMDM*1 in the microgametophyte development alone, we generated RNA interference (RNAi) transgenic plants of *AtNMDM1*, driven by the pollen-specific promoter, LAT52 (Figures 3A,B). In total, we obtained 86 T1 transgenic lines. Out of them, 48 T1 transgenic lines showed male fertility defects with a portion of the pollen stained green by Alexander’s staining. We randomly selected three transgenic lines, RNAi-4, RNAi-14, and RNAi-31 for further studies. The RT-qPCR data clearly showed that *AtNMDM1* transcript levels in anther stages 9–13 were greatly reduced in all the selected RNAi lines compared to that in the wild type (Figure 3C), indicating that the RNAi system worked well. Subsequently, these RNAi lines generated much shorter siliques and a large number of aborted pollen grains (Supplementary Figure 4 and Figure 3D), consistent with the previous data from genome-edited mutants (Figures 1C,E). In addition, the *AtNMDM1* RNAi lines had normal flowers and callose walls of the tetrads (Supplementary Figure 4), indicating that the interference of RNA was restricted to pollen. Therefore, the RNAi data further supported the function of *AtNMDM1* in the microgametophyte development.

### Pollen Defects Were Initiated at Anther Development Stage 10 in *AtNMDM1*^+ / −^ Mutants and *AtNMDM1* RNAi Lines

**FIGURE 4:**
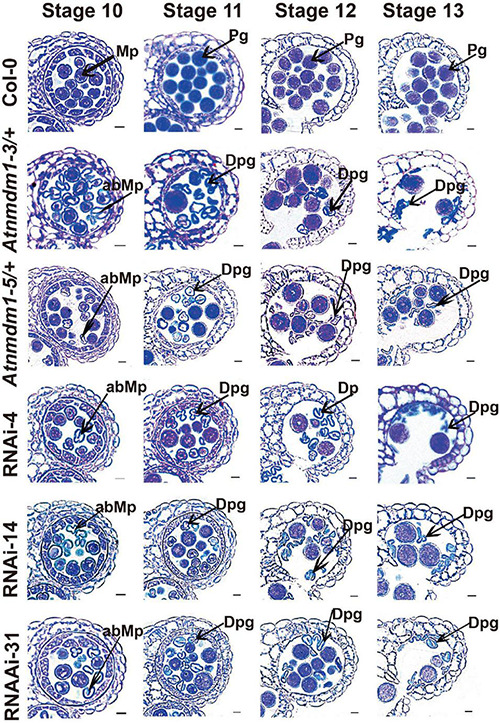
The *AtNMDM1*^+ / −^ mutants and RNAi lines were defective in pollen development. Semi-thin cross-sections of anthers from wild type, two *AtNMDM1*^+ / −^ mutants: *Atnmdm1-3/*+, *Atnmdm1-5/*+, and three *AtNMDM1* RNAi lines, RNAi-4, RNAi-14, and RNAi-31 were stained with toluidine blue. Anthers from stages 10–13 are shown. Microspores or pollen with reduced or completely lacking cytoplasm were defined as degenerate. Note that degenerate microspores were evident in two *AtNMDM1*^+ / −^ mutants: *Atnmdm1-3/*+, *Atnmdm1-5/*+ and three *AtNMDM1* RNAi lines RNAi-4, RNAi-14, and RNAi-31 from stages 10 to 13. Mp, microspore; abMp, abnormal microspore; Pg, pollen grain; Dpg, degenerated pollen grain. Bar = 10 μm.

To further investigate the detailed function of *AtNMDM1* in microgametophyte development, we examined the semi-thin sections of anthers from the *AtNMDM1*^+ / −^ mutants, *AtNMDM1* RNAi lines, and wild type plants. In the wild type, meiocytes underwent meiosis and generated meiotic product tetrads during anther stages 6 and 7. Newly formed microspores were released at stage 8 and became vacuolated at stage 9 (Supplementary Figure 5). In both *AtNMDM1*^+ / −^ mutants (*Atnmdm1-3/*+ and *Atnmdm1-5/*+) and *AtNMDM1* RNAi lines (RNAi-4, RNAi-14, and RNAi-31) of the same stages, the anthers appeared normal in size and shape, and the morphologies of the meiocytes, tetrads, and microspores were similar to those of the wild type (Supplementary Figure 5). During anther stages 10–12, the wild-type microspores underwent two rounds of mitotic divisions to form mature pollen, which contained two sperms and one vegetative cell (Figure 4). In contrast, the cytoplasm was shrunken, collapsed, and degraded in the microspores of *Atnmdm1-3*, *Atnmdm1-5*, and *AtNMDM1* RNAi lines (Figure 4). In addition, the abnormal microspores appeared to be less stained than those of the wild type (Figure 4). The cytoplasm of the abnormal microspores degenerated further, eventually resulting in aborted pollen at stage 13 (Figure 4). These results suggested that the defects of microspores after anther stage 10 ultimately led to male sterility in *AtNMDM1*^+ / −^ mutants and *AtNMDM1* RNAi lines.

### Pollen Intine Formation Is Disrupted in *AtNMDM1*^+ / −^ Mutants and *AtNMDM1* RNAi Lines

**FIGURE 5:**
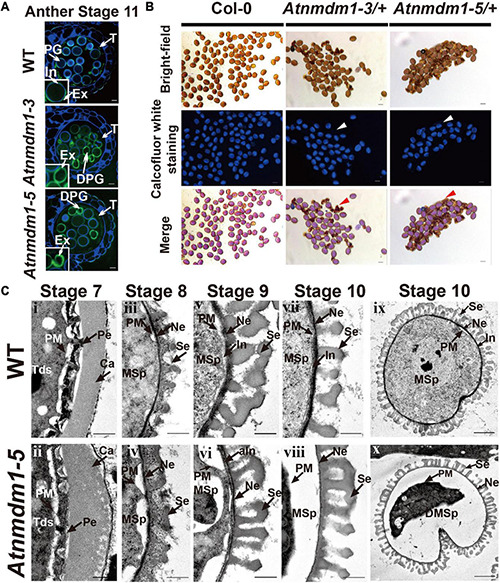
The development of the intine layer is abnormal in *AtNMDM1*^+ / −^ mutant*s*. **(A)** Cytochemical staining of semi-thin sections of wild type and *AtNMDM1*^+ / −^ mutants. All wild-type pollens showed a blue fluorescent ring of the intine layer while *Atnmdm1-3* and *Atnmdm1-5* pollen showed almost an absence of the intine layer. DPG, degenerated pollen grain; In, intine; Ex, exine; T, tapetum. Bar = 10 μm. **(B)** Calcofluor white staining of mature pollen in Arabidopsis. The pollen in the Col-0 and *Atnmdm1-3/*+, *Atnmdm1-5/*+ plants are shown. Arrowheads indicate the aborted pollen grains with no detectable fluorescence. Bar = 20 μm. **(C)** Transmission electron microscopy (TEM) observation of wild type and *Atnmdm1-5* pollen. TEM images of pollen walls at anther stages 7 and 8 showed that microspore layers were quite normal in the pollens of the wild type (i, iii) and *Atnmdm1-5* (ii, iv); (v, vi), stage 9, showing intine was abnormal in *Atnmdm1-5* pollen, compared to the wild type pollen; (vii, viii), stage 10, showing that the intine had almost disappeared in *Atnmdm1-5* pollen; (ix), stage 10, the content of the microspore in the wild type; (x), stage 10, showing the degenerated and condensed content in *Atnmdm1-5* microspores. Tds, tetrads; Ca, callose wall; PM, plasma membrane; Pe, primexine; MSp, microspore; Ne, nexine; Se, sexine; aIn, abnormal intine; DMsp, degenerated microspore. i–viii, bar = 500 nm; and, bar = 2 μm.

To further investigate the possible reasons for the pollen defects in *AtNMDM1*^+ / −^ (*Atnmdm1-3/*+ and *Atnmdm1-5/*+) and *AtNMDM1* RNAi lines (RNAi-4, RNAi-14, and RNAi-31), we examined the phenotypic defects in detail. Calcofluor white was used to detect the integrity of pollen intine at anther stage 11. The binding of Calcofluor white to cellulose, a major constituent of pollen intine, gives rise to a bright blue fluorescence under ultraviolet light excitation (Li et al., 2010). The wild-type pollen displayed a distinct fluorescent ring between the exine and cytoplasm, indicating the formation of an intact intine structure (Figure 5A). However, in the *Atnmdm1-3* and *Atnmdm1-5* pollens, the fluorescent ring of intine was hardly visible in the aborted pollens (Figure 5A), implying that the synthesis and/or the deposition of cellulose in the intine were impaired. In the *Atnmdm1-3/*+ and *Atnmdm1-5/*+ plants, we found two distinct types of mature pollens: one type appeared large and oval with intense fluorescence, like that in the wild type (Figure 5B); the other type was shrunken and wrinkled with no detectable fluorescence, suggesting that these pollens in *AtNMDM1*^+ / −^ plants lacked cellulose, a major component of intine (Figure 5B). We also observed abnormal intine formation in the aborted pollens of RNAi-4, RNAi-14, and RNAi-31 (Supplementary Figure 6). A dim and uneven fluorescence appeared between the exine and cytoplasm in the aborted pollens at stage 11 in the RNAi lines (Supplementary Figures 6C,F,I). These results demonstrated that knock-down of *AtNMDM1* could influence the normal intine formation during pollen development.

We then conducted a transmission electron microscopy (TEM) analysis to compare the morphologic differences between the wild type and *Atnmdm1-5* pollen. In *Atnmdm1-5* micropores, the structures of primexine and the callose wall at stage 7 (Figure 5C[ii]) and exine at stage 8 (Figure 5C) were similar to those of the wild type (Figures 5C[iii]). However, the intine of *Atnmdm1-5* pollen at stage 9 appeared to be abnormal and incomplete (Figure 5C[vi]) compared to the wild type (Figure 5C[v]). At stage 10, the intine of the abnormal pollen nearly completely disappeared, accompanied by the separation between the pollen cytoplasm and intine (Figure 5C[vi]). Compared to the wild type (Figure 5C[vii]), the basic structure of the exine layer was quite normal in *Atnmdm1-5* pollen (Figure 5C[viii]). From stages 10 to 12, the cytoplasm of pollen in the wild type was closely attached to the pollen wall, and the mature pollen contained intact and well-developed intine and extine layers (Figures 4, 5C). In contrast, the cytoplasm of *Atnmdm1-5*, the abnormal pollen was shrunken and degraded (Figure 5C[vii]). At stage 12, the pollen cytoplasm was further disintegrated, resulting in aborted pollen (Figure 4). In conclusion, the knock-out and knock-down of *AtNMDM1* had no effect on the exine development, but caused abnormal intine formation.

### Molecular Characterization and Subcellular Localization of *AtNMDM1*

**FIGURE 6:**
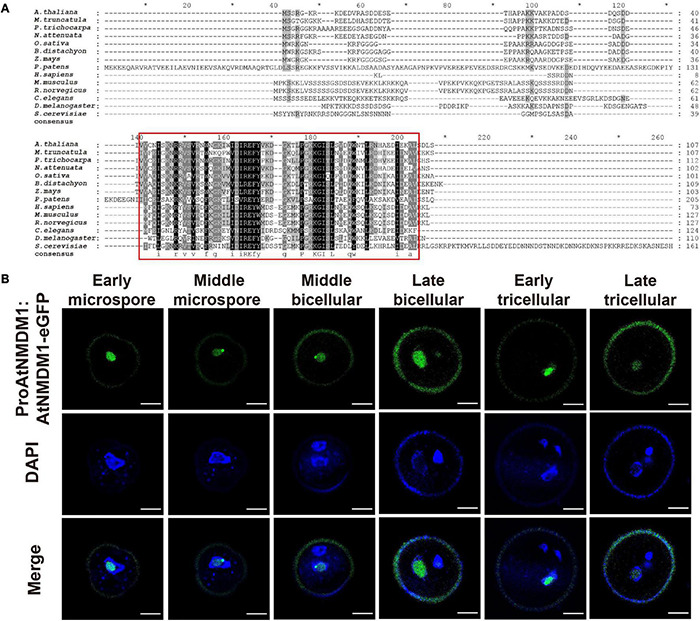
*AtNMDM1* encodes a conserved and nuclei-localized protein in pollen. **(A)** Multiple-sequence alignment of the *AtNMDM1* proteins from different species was performed by ClustalX. Conserved regions of *AtNMDM1* proteins are marked by red boxes. **(B)** Subcelluar localization of ProAtNMDM1:AtNMDM1-eGFP infusion protein during microgametogenesis. Representative pollen grains showed eGFP fluorescence (top, green), 4′,6-diamidino-2-phenylindole (DAPI) fluorescence (middle, blue), and eGFP and DAPI fluorescence merged (bottom) at early microspore, middle microspore, middle bicellular, late bicellular, early tricellular, and late tricellular stages. Bar = 5 μm.

*AtNMDM1* was predicted to encode a 107-amino acid (AA) protein with a molecular mass of 12.08 kilodaltons (kDa) and an isoelectric point (pI) of 6.73 (Supplementary Figure 7A). A transcriptional coactivator PC4-like region toward the C-terminal of the AtNMDM1 protein was visualized by using the NCBI Conserved Domain Search tool (Supplementary Figure 7A). Transcriptional coactivators, human PC4, and its yeast ortholog Sub1, were conserved in the PC4-like region with a single-stranded DNA-binding domain (ssDBD), which are involved in RNA polymerase II transcription (Ge and Roeder, 1994; Henry et al., 1996; Knaus et al., 1996; Garavis and Calvo, 2017). The BLAST search revealed that the PC4-like region exists widely in various species of plants, animals, and bacteria, implying a general biological function (Figure 6A). Sequence alignment identified a highly conserved region named PC4-like Region Conserved (PRC) located at the C terminus of the protein (Figure 6A), whereas the N-terminal region of AtNMDM1 homologs shared a low level of sequence similarity (Figure 6A). In addition, we found that nine obtained genome-edited mutated alleles of *AtNMDM1* were predicted to produce altered or truncated PRC domains (Supplementary Figure 7B). These results suggested that the PRC domain is potentially critical for the molecular function of *AtNMDM1*.

To investigate the subcellular localization of *AtNMDM1*, we analyzed transgenic plants carrying eGFP-infused AtNMDM1 construct driven by its native promoter (*ProAtNMDM1:AtNMDM1-eGFP*). We observed GFP signals in all the stages of the microspores and pollens (Figure 6B). In the early microspores, the GFP signals were intense in the nuclei; a relatively weak GFP signal was present in the middle stage microspores (Figure 6B). From middle bicellular pollen to early tricellular pollen, the GFP fluorescence increased in the nuclei of vegetative cells while it was merely detectable in the germline nuclei; the GFP signal in the nuclei of vegetative cells persisted but decreased in late tricellular pollen (Figure 6B). The GFP fluorescence at all stages appeared within the nuclei with light DAPI staining, but not where the DAPI signal was intense (Figure 6B). These results indicated that AtNMDM1 is a pollen nucleus localized protein, and it may be associated with transcriptional activity.

### AtNMDM1 Influences Intine Formation by Modulating the Expression of Related Genes

**FIGURE 7:**
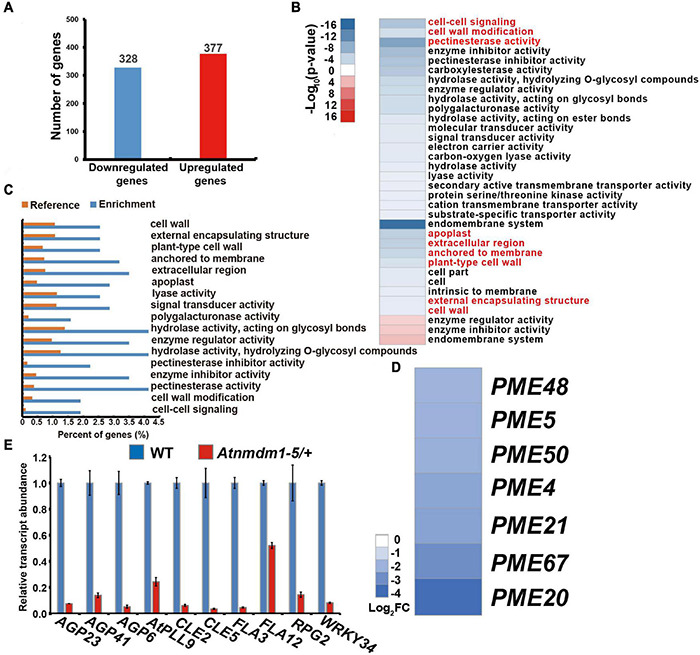
Transcriptome and RT-qPCR analysis of wild-type and *Atnmdm1-5/*+ stages 8 and 9 anthers. **(A)** The number of differentially expressed genes (DEGs) in *Atnmdm1-5/*+ relative to the wild type (log2FoldChange > 1 or log2FoldChange < -1, *P*-value > 0.05). **(B)** Heat map showing the functional annotation of genes with upregulated and downregulated expression in *Atnmdm1-5/*+ relative to the wild type. **(C)** Histogram showing the enrichment of genes with downregulated expression in *Atnmdm1-5/*+. **(D)** Heat map showing 7 pectin methylesterase (PMEs) genes with downregulated expression in *Atnmdm1-5/*+. **(E)** RT-qPCR analysis of intine and signal pathways associated with gene expression in the wild type and *Atnmdm1-5/*+ at the anther stages, 9 and 10. Data were normalized to *EF2*α expression. The bars represent mean ± SE of three biological replicates.

To investigate the role of AtNMDM1 in transcription activity, a high-throughput whole-transcriptome sequencing (RNA-Seq) was implemented on both wild type and *Atnmdm1-5/*+ stage 8 and stage 9 anthers. Differential gene expression analyses revealed that 328 genes showed downregulated expression and 377 genes had upregulated expression in *Atnmdm1-5/*+ anthers, compared to those in the wild type (*P* < 0.05, log2 (Fold Change) > 1 or log2 (Fold Change) < −1) (Figure 7A and Supplementary Data Set 2). AgriGO analysis revealed that clustering was mainly concentrated on 31 categories for the downregulated genes, while only three categories were enriched for the upregulated genes (*P* < 0.05) (Figure 7B), implying that AtNMDM1 mainly acts as a positive regulator in gene expression.

We then performed a GO term analysis and enrichment of the GO terms of the downregulated genes alone. In terms of biological processes, cell–cell signaling and cell wall modification categories were significantly enriched (Figures 7B,C and Supplementary Data Set 3). With respect to molecular functions, pectin esterase activity, enzyme inhibitor activity, hydrolase activity on hydrolyzing O-glycosyl compounds, enzyme regulator activity, hydrolase activity acting on glycosyl bonds, polygalacturonase activity, lyase activity, and signal transducer activity were significantly enriched (Figures 7B,C and Supplementary Data Set 3). Regarding cellular components, the proteins corresponding to the downregulated genes were predicted to be mainly located at the apoplast, the extracellular region anchored to the membrane, plant-type cell walls, and in the external encapsulating structure cell walls (Figures 7B,C and Supplementary Data Set 3). Therefore, GO term analysis revealed a strong enrichment in several categories representing genes involved in cell-wall development and signaling pathways, consistent with the abnormal pollen intine in *AtNMDM1*^+ / −^ mutants and *AtNMDM1* RNAi lines (Figure 5 and Supplementary Figure 6).

To verify the reduced gene expression related to cell-wall formation and signaling pathway in *Atnmdm1-5/*+, we isolated anthers at stages 8 and 9 from both the wild type and *Atnmdm1-5/*+ for RT-qPCR analysis. Consistent with the RNA-seq data, cell wall-associated genes (*AGP6, AGP23, AGP41, AtPLL9, FLA3, FLA12, RPG2*) and signaling pathway genes (*CLE2, CLE5, WRKY34*) were significantly downregulated in *Atnmdm1-5/*+ compared to the wild type (Figure 7E and Supplementary Data Set 4). In particular, the fasciclin-like arabinogalactan protein gene *FLA3*, which has been shown to be involved in pollen intine development (Li et al., 2010), was greatly reduced (Figure 7E), suggesting the role of arabinogalactan protein in intine development. Pectin methylesterases are hydrolytic enzymes in plants, which strengthen or relax plant cell walls by remodeling and decomposing the pectin (Bosch et al., 2005; Pelloux et al., 2007). Specifically, PME48 affects the mechanical properties of the intine by remodeling the pectins in the pollen of *Arabidopsis* (Leroux et al., 2015). Our RNA-seq data showed that seven *PMEs* genes, such as *PME48*, were significantly downregulated (Figure 7D and Supplementary Data Set 4). Thus, *AtNMDM*1 impacts intine formation by regulating the expression of intine-related genes, such as *AGPs* and *PMEs*.

### The Identification of AtNMDM1 Interacting Proteins by Immunoprecipitation Mass Spectrometry

To identify proteins interacting with AtNMDM1, an IP/MS was performed, in which young inflorescence tissues carrying *ProAtNMDM1:AtNMDM1-3* × *FLAG* construct was used. In total, we identified 802 peptides, which corresponded to 567 distinct peptides on 247 proteins (Supplementary Data Set 5). The length of the identified protein ranged from 64 to 2,355 amino acids, with 99% falling within the range of 66–990 amino acids (Supplementary Figure 8A). The number of distinctly identified peptides per protein was positively correlated with the number of identified peptides per protein (Supplementary Figure 8B). Here, the value of peptide spectrum match (PSM) was the total number of identified peptide sequences for the protein, including those redundantly identified. AtNMDM1 possessed 69 PSMs, which were ranked Number 1 according to the value of PSMs, indicating the high reliability of the IP/MS data (Supplementary Data Set 5). In addition, we found that only AT4G12960, a gamma-interferon-responsive lysosomal thiol reductase, was detected in both IP/MS and RNA-seq data (Supplementary Figure 8C).

To better understand the potential functions of the identified proteins, we firstly performed GO enrichment analysis using the AgriGO tool (Tian et al., 2017). The GO lists were further summarized with the REVIGO tool for reducing functional redundancies (Supek et al., 2011). Forty non-redundant GO terms were statistically enriched (*P* < 0.01, dispensability <0.5), consisting of biological processes, cellular components, and molecular functions (Supplementary Figures 8D,F). For biological processes, 16 GO terms, including cellular process, nucleoside metabolic process, nucleobase-containing small-molecule metabolic process, and translation, were significantly enriched (Supplementary Figure 8D). Regarding the cellular component, we found that those proteins were largely assigned to macromolecular complexes, cytoplasm, and intracellular and non-membrane-bounded organelles (Supplementary Figure 8E). As for the molecular function, proteins associated with structural molecule activity, structural constituent of ribosome, and RNA binding and translation factor activity were highly enriched (Supplementary Figure 8F). These results implied that AtNMDM1 may be involved in important cellular processes, such as nucleoside metabolic process and translation.

Notably, among those processes, three proteins associated with RNA polymerase II transcription, transcription factor IIB (TFIIB1, AT2G41630), polypyrimidine tract-binding protein 1 (PTB1, AT3G01150), and DNA-directed RNA polymerase family protein (AT2G15400), were identified (Supplementary Data Set 5). Furthermore, arginine/serine-rich zinc knuckle-containing protein 33 (AT2G37340) and ssDNA-binding transcriptional regulator (AT2G02740), likely functioning as transcription factors, were possibly related to the regulated pollen wall gene transcription (Supplementary Data Set 5). Moreover, we noticed that four WD-40 domain proteins, namely AT1G15750, AT1G71840, AT1G29320, and AT3G49400 may function as a protein–protein or protein–DNA interaction platform in a large majority of cellular processes (Supplementary Data Set 5). Considering that these processes and the identified proteins were highly related to translation activity, we hypothesized that AtNMDM1 may be involved in the mechanism of RNA polymerase II transcription and mRNA processes.

### AtNMDM2 Interacting With AtNMDM1 in Pollen Nuclei

In our IP/MS data, we noticed that AT4G10920, also annotated as a transcriptional coactivator, was identified to potentially interact with AtNMDM1 (Supplementary Data Set 5), implying that these two proteins might form a heterodimer to perform their functions. We named AT4G10920 as AtNMDM2 for convenience, and AtNMDM2 is used below.

**FIGURE 8:**
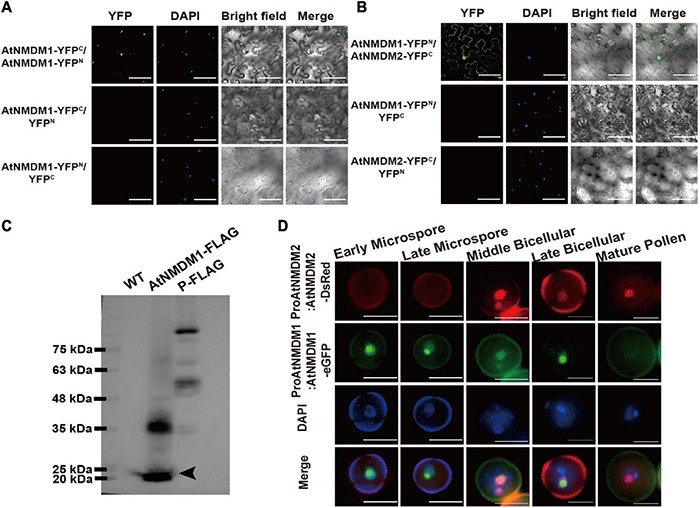
AtNMDM1 interacts with self and AtNMDM2. **(A)** Examination of homodimers of AtNMDM1 by BiFC assay. **(B)** BiFC visualization of the interaction between AtNMDM1 with AtNMDM2. Construct pairs showed at the left were cotransformed in the leaves of *N. benthamiana* by agroinfiltration. Nuclei are indicated by DAPI staining. Yellow fluorescent protein (YFP) signals were detected and imaged by confocal microscopy. Left panels, YFP signal images. Middle panels, DAPI fluorescence, and bright-field images. Right panels, merged DAPI fluorescence images with YFP signals and bright-field images. These experiments were repeated at least three times with similar results. Bars in **(A,B)** = 100 μm. **(C)** Western blot of AtNMDM1 protein infused with FLAG tag. Proteins extracted from the young inflorescence were separated by SDS-PAGE. The protein marker is indicated in the left panel. No bands are shown in the panel of the wild type (WT). Transgenic plants containing AtNMDM1 infused protein with FLAG tag accumulated a putative monomeric protein at approximately 20 kDa (black arrow) while a putative ∼40 kDa dimer was also present in the panel of AtNMDM1-FLAG. P-FLAG is a 104-kDa- infused protein. The panel of P-FLAG acts as the positive control. **(D)** AtNMDM1 colocalizes with AtNMDM2 in pollen nuclei. AtNMDM1-eGFP fusion protein was coexpressed with AtNMDM2-DsRed in the pollen. Fluorescence signals were observed by fluorescence microscopy. From top to bottom, representative pollen grains show DsRed fluorescence (top, red), eGFP fluorescence (next to top, green), DAPI fluorescence (middle, blue), and the three above mentioned fluorescences merged (bottom) at early microspore, late microspore, middle bicellular, late bicellular, and mature pollen grain stages. Bar = 10 μm.

To test whether AtNMDM1 interacted with AtNMDM2 in plant cells, we performed a bimolecular fluorescence complementation (BiFC) experiment. No fluorescence was detectable with negative control-combinations of AtNMDM1-YFP*^C^*/YFP*^N^*, AtNMDM1-YFP*^N^*/YFP*^C^*, and AtNMDM2-YFP*^C^*/YFP*^N^* (Figures 8A,B). Reconstituted YFP fluorescence in the nuclei was detected with experimental combinations of AtNMDM1-YFP*^C^*/AtNMDM1-YFP*^N^* and AtNMDM1-YFP*^N^*/AtNMDM2-YFP*^C^*, indicating that AtNMDM1 interacted with itself and with AtNMDM2 in the nuclei, respectively (Figures 8A,B). Furthermore, we performed Western Blot using protein extracts from the inflorescence of the wild type and the transgenic plants with the *ProAtNMDM1:AtNMDM1-FLAG* fragment. We found that transgenic plant inflorescences accumulated a putative monomeric AtNMDM1 infused protein at approximately 20 kDa (Figure 8C). Another intense band appeared at around 40 kDa (Figure 8C), corresponding to the molecular weight of an AtNMDM1 dimer; in comparison, no bands were detected for the wild type (Figure 8C). These results implied that AtNMDM1 functions as a dimer with itself or with AtNMDM2.

To further investigate whether AtNMDM1 and AtNMDM2 are co-localized in the pollen, we crossed the transgenic Arabidopsis *ProAtNMDM1:AtNMDM1-eGFP* with plants carrying the *ProAtNMDM2:AtNMDM2-DsRed* (*Discosoma* red fluorescent protein) construct. In the microspore stage, no DsRed signal was detectable (Figure 8D). However, an intense AtNMDM2-DsRed signal was observed in the nuclei at the middle bicellular pollen stage, which was stronger in the vegetative nuclei than the reproductive nuclei (Figure 8D). In the late bicellular stage, the AtNMDM2-DsRed signal started to decline, remaining weakly detectable in the vegetative nuclei at the late bicellular and mature pollen stage (Figure 8D). In contrast, an intense AtNMDM1-eGFP signal was seen in the nuclei of microspores and bicellular pollen grains but not in the mature pollen, consistent with the previous data (Figures 8B,D). We observed that the GFP signals colocalized with DsRed signals in the nuclei of bicellular pollen grains (Figure 8D). These results indicated that AtNMDM1 and AtNMDM2 mainly colocalize in the nucleus at the bicellular pollen stage.

## Discussion

### *AtNMDM1* Plays an Important Role in Pollen Intine Development

Intine is the inner layer of the pollen wall, which is present between the plasma membrane and the exine. Formation of the intine begins when the microspores are released from tetrads; this occurs later than the development of the exine (Owen and Makaroff, 1995). The intact intine is important for the survival and fertility of pollen, assuring the viability of mature pollen as well as pollen-tube germination (Edlund et al., 2004; Schnurr et al., 2006). Only a few genes involved in intine development, including *AtUSP*, *CESA3 FLA3*, and *PME48*, have been identified in Arabidopsis (Drakakaki et al., 2006; Schnurr et al., 2006; Li et al., 2010; Leroux et al., 2015). This might be due to the difficulty of observation of the relevant defects in the intine. Therefore, the molecular mechanisms controlling intine development remain largely unknown, especially at the regulatory level of gene expression. Here, we identified that *AtNMDM1* was functionally involved in intine development. *AtNMDM1* encoded a putative transcriptional coactivator, in agreement with its localization in the nuclei of pollen grains (Figure 6B). It has been reported that *AtNMDM1* is highly expressed in inflorescence (Cormack et al., 1998). Transcriptome analysis of pollen grains shows that the expression of *AtNMDM1* is detected in haploid microgametophytes (Honys and Twell, 2004). We further showed that *AtNMDM1* was highly expressed in the pollen at anther stages 10–12 and could not be detected at stage 13 (Figures 2F–I). The knockout and knockdown of *AtNMDM1* greatly affected the pollen development without affecting the development of the vegetative tissues, thus resulting in a lower seed-setting rate (Figures 1C–E, 3D, Supplementary Figures 2E,4, and Supplementary Table 2). Moreover, analyses of Calcofluor white staining and TEM clearly demonstrated that intine pollen was abnormal in both *AtNMDM1*^+ / −^ and *AtNMDM1* RNAi plants, leading to gametophytic lethality (Figure 5 and Supplementary Figure 6).

In general, intine development is mainly determined by microgametophytes (Van Aelst et al., 1993; Shi et al., 2015). In our results, we found that sporophytic anther walls appeared normal in both *AtNMDM1*^+ / −^ and *AtNMDM1* RNAi plants following the observation of the semi-thin sections of the anther (Figure 4 and Supplementary Figure 5), suggesting that intine formation is indeed regulated by microgametophytes. In the analyses of genetic transmission, we discovered that *Atnmdm1-3* and *Atnmdm1-5* alleles were mainly delivered by female gametophytes (Supplementary Table 1). Those results strongly suggested that *AtNMDM1* is a microgametophyte lethal gene. A stable genetically homozygous line with microgametophyte defects is not available. However, when the pollen from an *Atnmdm1-5/*+ plant was the male donor, 47% of their progeny possessed the abnormal pollen phenotype. This result was not consistent with the Mendelian theory, in which the phenotype of all F2 plants should be similar to the wild type (Supplementary Table 1). Given that the Cas9/gRNA system is not eliminated in *Atnmdm1-5/*+, we hypothesize that this system is transmitted to the next generation by viable pollen in the *Atnmdm1-5/*+ plant and continues to edit *AtNMDM1*.

We also found that normal-type pollen was viable and female fertility was reduced in the reciprocal cross-analysis of *AtNMDM1*^+ / −^ mutants (Supplementary Table 2). This result is consistent with the high transcript level of *AtNMDM1* in the pistils at various stages (Figures 2B,C). Furthermore, the knock-down of the *AtNMDM1* transcript level resulted in pollen intine defects (Figure 3D), supporting the role of AtNMDM1 functioning in intine development. However, intine defects of *AtNMDM1*^+ / −^ mutants were more severe than those of *AtNMDM1* RNAi lines (Figure 5 and Supplementary Figure 6), presumably owing to the usage of *LAT52* promoter in the RNAi system, which were mainly effective in the bicellular and tricellular stages of pollen development (Twell et al., 1990; Eady et al., 1994). In conclusion, *AtNMDM1* is a microgametophyte lethal gene that functions as a new regulator involved in the development of pollen intine, which affects the normal development of microgametophytes.

### AtNMDM1, Together With Various Regulators, May Be Important Components of a Complicated Transcriptional Complex

In eukaryotes, RNA polymerase II is primarily responsible for the transcription of protein-coding genes and small non-coding RNAs (Turowski, 2013). Coactivators are important components of the RNAPII complex (Thomas and Chiang, 2006). In humans, the transcriptional coactivator, PC4 is required to stimulate the reconstituted basal transcription by RNA polymerase II *in vitro* (Werten et al., 1998). PC4 is thought to be evolutionarily conserved in an ssDBD (Werten et al., 2016; Garavis and Calvo, 2017). Here, we found that a PRC region (PC4-like Region Conserved) in AtNMDM1 was highly conserved in plants, humans, and yeast (Figure 6). Altered or truncated PRC domains in *AtNMDM1*^+ / −^ mutants led to fertility defects (Figure 1, Supplementary Table 2, and Supplementary Figure 7). Therefore, we speculated that the PRC domain is important for the AtNMDM1 transcriptional function, which is likely to be responsible for AtNMDM1 binding to ssDNA.

Gene Ontology analysis of IP/MS data indicated that the identified proteins were enriched in the categories of cellular process and nucleoside metabolic process (Supplementary Figure 8D), implying the potential function of AtNMDM1 related to transcriptional activity. The IP/MS data first revealed another putative transcriptional coactivator, AtNMDM2, which was highly abundant in the inflorescence (Supplementary Data Set 5). It has been reported that AtNMDM1 interacts with AtNMDM2 *in vitro* (Cormack et al., 1998). Here we demonstrated that AtNMDM1 interacted with AtNMDM2 in the nuclei of plant cells *in vivo* (Figure 8B); AtNMDM1 colocalized with AtNMDM2 in the nuclei of pollen, suggesting their role in pollen development (Figure 8D). In yeast, the homolog of PC4 (Sub1) interacted with TFIIB, forming key parts of the RNAP II complex (Knaus et al., 1996; Wu et al., 1999). Moreover, AtNMDM1 interacted with AtNMDM2 in the cytoplasm of plant cells (Figure 8B). According to previous studies, AtNMDM2 is also present in the cytoplasm of epidermal cells in *Nicotiana benthamiana* and is involved in the movement protein (MP) function of cell-to-cell movement of virus through its interaction with MP encoded by Tomato mosaic virus *in vivo* (Sasaki et al., 2009). These results suggested that AtNMDM1 and AtNMDM2 may also be involved in the cell-to-cell movement of the Tomato mosaic virus.

In the IP/MS data, we identified a transcription factor, AtTFIIB1 (AT2G41630) (Supplementary Data Set 5). It has been reported that AtTFIIB1 is involved in the growth of pollen tube and endosperm development, but it is located in pollen nuclei at different stages, especially in the vegetative nuclei at the tricellular pollen stage (Zhou et al., 2013); the subcellular localization pattern is similar to that of AtNMDM1 (Figure 6B). Thus, we speculated that AtNMDM1 may interact with AtTFIIB to participate in the assembly of RNA polymerase II complex and mRNA transcription processes. WD40 domain proteins mainly function as a protein–protein or protein–DNA interaction platform in a large majority of cellular processes (Xu and Min, 2011). Four WD40 domain proteins, such as AT1G15750, AT1G71840, AT1G29320, and AT3G49400, were identified in IP/MS (Supplementary Data Set 5). In particular, the WD-40 domain protein, AT1G15750 (TOPLESS), a corepressor, plays a negative role in BRI1-EMS-SUPRESSOR 1 (BES1)-mediated brassinosteroid-induced transcriptional regulation (Espinosa-Ruiz et al., 2017). It is possible that the four identified WD40 proteins function as a protein–DNA interaction platform in the transcription activity of RNA polymerase II during pollen development. Our RNA-seq data and RT-qPCR analysis indicated that the expression of *WRKY34*, a gene involved in signaling pathways, was significantly downregulated in *Atnmdm1-5*/+ (Figure 7E and Supplementary Data Set 4). The pollen-specific gene *WRKY34* encodes a nuclei-localized transcription factor, which negatively regulates the sensitivity of mature pollen to cold temperatures (Zou et al., 2010). Our transcriptome analysis suggested that WRKY34 may be involved in the transcriptional complex together with AtNMDM1. As a result, the transcriptional complex mediated by AtNMDM1 seems rather complicated, and more regulatory factors await to be identified and verified.

### AtNMDM1 Plays Essential Roles in Intine Development by Regulating the Expression of Arabinogalactan Proteins and Pectin Methylesterase Genes

The major constituents of intine include cellulose, hemicellulose, pectin, and various structural proteins, such as arabinogalactan proteins (AGPs) (Knox, 1971; Knox and Heslop-Harrison, 1971; Aelst and Went, 1992). Calcofluor white staining and TEM analysis showed that intine cellulose was still deposited during the early developmental stages of microspores in *Atnmdm1-3* and *Atnmdm1-5* alleles (Figure 5 and Supplementary Figure 6). Our RNA-seq data indicated the expression of cellulose synthesis-related genes, such as *AtUSP*, *CESA1*, and *CESA3*, which were normal in *Atnmdm1-5/*+ as well as in the wild type (Supplementary Data Set 4). However, the transcripts of *AGP* genes, including *FLA3, FLA12, AGP6, AGP23*, and *AGP41*, were significantly downregulated (Figure 7E). AGPs are a class of highly glycosylated hydroxyproline-rich glycoproteins, that are widely found on the extracellular surface of plant cells and are thought to be involved in many biological processes (Showalter, 2001; Ellis et al., 2010). It is known that FLA3 (a fasciclin-like arabinogalactan protein) affects cellulose deposition and the formation of pollen intine (Li et al., 2010). The double mutant, *agp6 agp11* has defects in pollen fertility and pollen tube growth (Levitin et al., 2008; Coimbra et al., 2009). Knockout of *AtFLA11* and *AtFLA12* leads to altered cell wall composition with reduced arabinose, galactose, and cellulose contents (Ito et al., 2005; MacMillan et al., 2010). It has been shown that normal deposition of the intine is dependent on an intact nexine, while AGP6, AGP11, AGP23, and AGP40 are suspected to be important components for the nexine layer (Jia et al., 2015). Pollen transcriptome data of the wild type indicated that *AGP6* and *AGP23* were highly expressed in the pollen at different stages (Supplementary Data Set 6) q2 (Honys and Twell, 2004). OsUCL23, a chimeric AGP, is involved in the transport and accumulation of metabolite flavonoids and thus regulates pollen intine development (Zhang et al., 2020). Given this evidence, AGPs may play a key role in the development of intine and AtNMDM1 may influence intine development by regulating the expression of the *AGPs* gene.

Pectin is an important component of pollen intine. In Arabidopsis, pectin methylesterase 48 (PME48) affects the mechanical properties of pollen intine by regulating the degree of methyl esterification of homogalacturonan, a major component of pectin, which then impacts pollen germination (Leroux et al., 2015). Our RNA-seq data indicated that GO term pectinesterase activity was significantly enriched in downregulated genes (Figures 7B,C); in particular, seven *PMEs* including *PME48* were significantly downregulated (Figure 7D and Supplementary Data Set 4). In *Brassica campestris*, pectin methylesterases, such as BcMF23a and BcPME37c, are involved in pollen intine formation (Yue et al., 2018; Xiong et al., 2019). This implies that the effects of pectin methylesterases on pollen intine development are universal. Our transcriptome and RT-qPCR analyses revealed that the transcript of *pectate lyase-like 9* in Arabidopsis (*AtPLL9*) was significantly reduced in *Atnmdm1-5/*+ (Figure 7E and Supplementary Data Set 4); The homologous gene of *AtPLL9* in *Brassica campestris BcPLL9* plays a crucial role in intine formation (Jiang et al., 2014b) which suggests that pectin in *Atnmdm1* pollen might be also affected.

In conclusion, except for regulating the transcript level of *AGPs*, AtNMDM1 also regulates the expression of *PMEs* and *AtPLL9*, which may affect the pectin structure of the intine and thus impact pollen intine development.

### The Model of AtNMDM1 Regulating Intine Development

In this study, the knockdown and knockout of AtNMDM1 led to abnormal cellulose distribution in the pollen intine (Figure 5 and Supplementary Figure 6). The transcript level of *AGPs* and *PMEs* were significantly downregulated in *AtNMDM1*^+ / −^ mutant (Figures 7D,E and Supplementary Data Set 4). The relationship between cellulose, hemicellulose, pectin, and AGPs in pollen intine is unknown. According to recent reports, FLA3 and AGP6 with a glycosylphosphatidylinositol (GPI) anchor are located at the cell membrane and pollen wall (Li et al., 2010; Pereira et al., 2013). In the fractionated cell walls of Arabidopsis, AGP-associated carbohydrate epitopes are enriched with extracted pectin and hemicellulose (Moller et al., 2007). AGPs can form a covalent link with both hemicellulose and pectin. In particular, rhamnogalacturonan I (RG I) or homogalacturonan (HG) types of pectin, are linked to the rhamnosyl residue in arabinogalactan (AG) of the AGP to form a covalent bond (Tan et al., 2013). Furthermore, AGPs link with pectic polysaccharides depending on calcium ion-driven electrostatic interaction in English ivy (Huang et al., 2016). Furthermore, neutral pectin polysaccharides can bind to cellulose *in vitro* (Zykwinska et al., 2005). Taken together, this suggested that AGPs may indirectly act on the distribution of cellulose by linking with the pectin. Therefore, we surmised that the mutation of *AtNMDM1* led to a decrease in the amount of AGPs and an abnormal modification of pectin, which in turn affected the binding of pectin to AGPs. Subsequently, cellulose was abnormally distributed, which finally resulted in abnormal intine formation.

How does abnormal intine cause the abortion of pollen? Programmed cell death (PCD) is the process of controlled cellular suicide, being crucial in the development of multicellular organisms as well as in the removal of damaged or infected cells (Lam, 2004). The process of apoptotic PCD-like tapetal degeneration is characterized by cellular condensation, mitochondrial and cytoskeletal disintegration, and DNA fragmentation (Parish and Li, 2010). Collapsed pollen grains undergoing PCD are caused by the abnormal pollen wall in the mutants defective in tapetal degeneration (Phan et al., 2011). In plants, reactive oxygen species (ROS) may function as signaling molecules, opening the permeability transition pore in mitochondria and releasing cytochrome *c*. Consequently, a greater accumulation of ROS finally results in cell PCD (Reape and McCabe, 2010). Recent evidence shows that AGPs can bind and release apoplastic calcium through glucuronidated arabinogalactan polysaccharides (Lopez-Hernandez et al., 2020). This implies that AGPs may also act as extracellular signal transduction messengers to transmit other signals generated by PCD. In our results, the cytoplasm of microspores was shrunken, collapsed, and degraded, which was caused by the abnormal pollen wall in the *AtNMDM1*^+ / −^ and *AtNMDM1* RNAi lines (Figures 4,5 and Supplementary Figure 6); The characteristics of the above-mentioned microspore apoptosis are quite similar to those of PCD. Thus, we hypothesized that ROS was generated by abnormal deposition of intine leading to PCD in aborted pollen.

**FIGURE 9:**
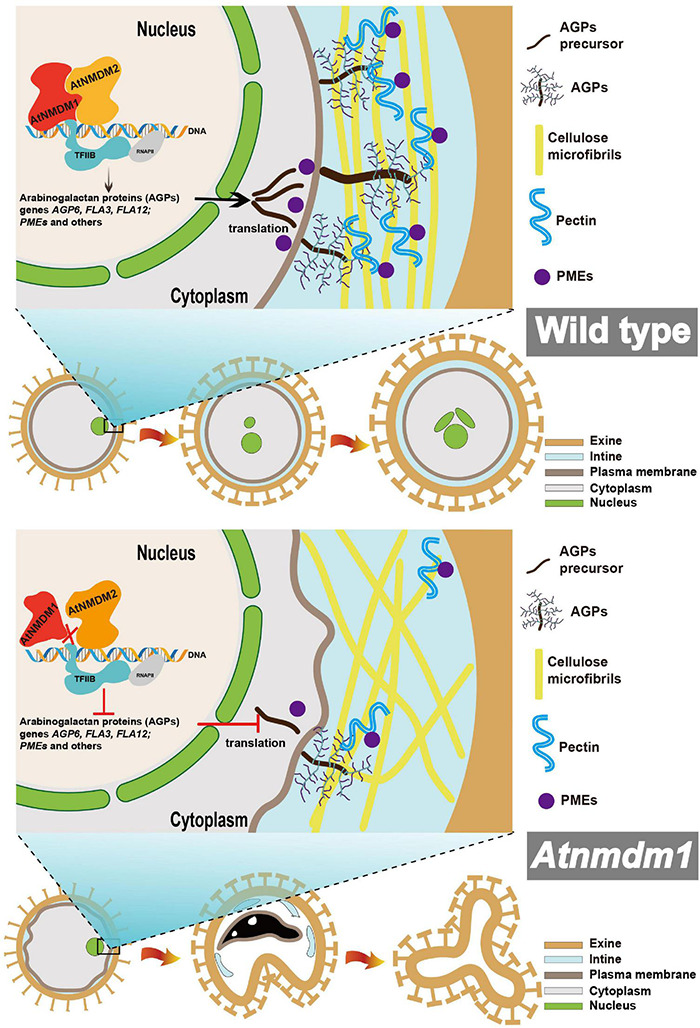
Model of the proposed role for AtNMDM1 regulating AGP and PME genes and thus affecting intine development. AtNMDM1 forms an RNAP II transcription complex together with AtNMDM2 and transcription factor II B (TFIIB), regulating the expression of AGPs and PMEs. AGPs and PMEs function together in the development of the pollen wall, leading to a normal distribution of cellulose, then resulting in the abnormal development of the pollen intine layer. The dashed line represents the potential pathway. ROS, reactive oxygen species; RNAP II, RNA polymerase II; AGPs, arabinogalactan proteins; PMEs, pectin methylesterase; PCD, programmed cell death.

According to our data, we proposed a model for illustrating the function of AtNMDM1 in pollen development (Figure 9). In wild-type pollen, AtNMDM1 forms an RNAP II transcription complex together with AtNMDM2 and transcription factor II B (TFIIB), promoting the expression of *AGPs* and *PMEs*. The AGPs are delivered to the intine and then may bind to pectins to maintain the normal distribution of cellulose microfibrils, ensuring the normal development of intine and pollen. In *Atnmdm1* pollen, the mutated AtNMDM1 fails to bind with AtNMDM2 and transcription factor IIB, leading to the production of aberrant RNAPII transcription complexes, consequently reducing the expression of *AGPs* and *PMEs*. Therefore, total amounts of AGPs and PMEs in the intine are decreased and the pectin cannot be correctly modified and arranged normally, leading to abnormal distribution of cellulose microfibrils. As a result, the impaired intine causes the production of ROS signals that trigger the PCD of pollen, eventually leading to pollen abortion.

## Data Availability Statement

The data presented in the study are deposited in the NCBI BioProject database repository, accession number PRJNA797722.

## Author Contributions

LM performed most of the experiments and data analyses wrote a draft for the manuscript. AM, JY, HuL, DR, and WC assisted with the phenotypic analysis. HaL assisted with the generation of the mutants. NJ performed bioinformatics analysis for transcriptome sequencing. TZ provided valuable advice and modified the written manuscript. PL conceived the study, designed the experiments, interpreted the data, supervised the project, and modified the manuscript. All authors contributed to the article and approved the submitted version.

## Conflict of Interest

The authors declare that the research was conducted in the absence of any commercial or financial relationships that could be construed as a potential conflict of interest.

## Publisher’s Note

All claims expressed in this article are solely those of the authors and do not necessarily represent those of their affiliated organizations, or those of the publisher, the editors and the reviewers. Any product that may be evaluated in this article, or claim that may be made by its manufacturer, is not guaranteed or endorsed by the publisher.

## Footnotes

1
http://www.genome.arizona.edu/crispr/

2
https://www.ncbi.nlm.nih.gov/tools/primer-blast/

3
http://biodb.swu.edu.cn/qprimerdb

4
https://www.arabidopsis.org/

5
https://www.ncbi.nlm.nih.gov/Structure/cdd/wrpsb.cgi

6
https://www.ncbi.nlm.nih.gov/

7
https://megasoftware.net/download_form

8
https://www.ncbi.nlm.nih.gov/bioproject/

## Supplementary Material

Supplementary Figure 1 Examples of direct sequencing PCR products containing targeted site 1 of *AtNMDM1* in T1 transgenic plants. **(A)** The genomic structure of the *AtNMDM1* from Arabidopsis. Exons are represented by black boxes. Introns are shown by a dashed line. The target site1 is indicated by a red arrow. The length of *AtNMDM1* is 1344 bp. **(B)** The sequencing chromatograms with overlapping traces are indicated at the target site1 of *At5g09250* by using the direct sequencing method in T1 individuals. The wild-type sequence is shown at the top with the PAM sequence highlighted in blue and the target sequence in red. **(C)** The target site1 of *At1g77250* was not edited.

Supplementary Figure 2 New variants of *AtNMDM1* were generated in T2 transgenic plants; and examples of sequencing chromatograms containing targeted sites, using a complex-sequencing method in T2 transgenic plants. **(A,C)** New mutations at *AtNMDM1* were generated in T2 transgenic plants. The wild-type sequence is shown at the top with the PAM sequence highlighted in blue and the target sequence in red. Green dash and letter indicate deleted bases and insertions or changed bases, separately (+, insertion; −, deletion; letter-letter, changed); New variants are shown in red boxes. **(B,D)** Examples of sequencing chromatograms. Wild type allele and *Atnmdm1-3* allele at the targeted site 1 **(B)** and wild type allele and *Atnmdm1-5* allele at the targeted site 2 **(D)** are shown using a complex-sequencing method. Red arrows show the start positions where, or from where, the mutations occurred. PAM is shown by a black underline. Black arrows indicate the corresponding start positions from where the mutations occurred in the wild type. **(E)** Vegetative growth of wild type and *AtNMDM1*^+ / −^ mutants, *Atnmdm1-3/*+, and *Atnmdm1-5/*+ were quite normal in the plants after sowing at 39 or 40 days. Bars = 2 cm (left), 1 cm (right).

Supplementary Figure 3 *AtNMDM1* promoters trigger GUS expression during the growth stage in Arabidopsis. **(A–C)** show 1–14 day Pro*AtNMDM1*:GUS transgenic seedlings. **(D)** Young siliques after pollination. **(E)** Embryo in a young silique. **(F)** Different stages of siliques. GUS expression in transgenic plants carrying Pro*AtNMDM1*: GUS is indicated in blue following staining for GUS activity. **(A–D,F)**, bar = 1 mm; **(E)**, bar = 500 μm.

Supplementary Figure 4 Analysis of the phenotype of *AtNMDM1* RNAi lines. From top to bottom, the callose wall in *AtNMDM1* RNAi lines RNAi-4, RNAi-14, and RNAi-31 are quite normal around the microspores compared with the wild type ones (top); the number of pollen grains in *AtNMDM1* RNAi lines was less than in the wild type (middle); the silique length of *AtNMDM1* RNAi lines was obviously shorter than in the wild type (bottom). Callose wall, bars = 10 μm. Opened flower, bars = 1 mm. Silique, bars = 5 mm.

Supplementary Figure 5 The phenotype of *AtNMDM1*^+ / −^ mutants and RNAi lines were normal-type in anther stages 6–9. Semi-thin cross-sections of anthers from wild type, *Atnmdm1-3/*+, *Atnmdm1-5/*+ and three *AtNMDM1* RNAi lines RNAi-4, RNAi-14, and RNAi-31 were stained with toluidine blue. The microspores were quite similar for the wild type in *Atnmdm1-3/*+, *Atnmdm1-5/*+, and three *AtNMDM1* RNAi lines—RNAi-4, RNAi-14, and RNAi-31—from stages 6 to 9. T, Tapetum; MMC, microspores mother cell; Tds, tetrads; Mp, microspore. Bars = 10 μm.

Supplementary Figure 6 Calcofluor white staining of semi-thin sections of *AtNMDM1* RNAi lines. **(A,D,G)** present anther sections at stage 11 in the *AtNMDM1* RNAi lines RNAi-4, RNAi-14, and RNAi-31, respectively. Calcofluor white staining revealed that all normal-like pollen showed a blue, fluorescent ring of the intine layer in the RNAi-4 **(B)**, RNAi-14 **(E)**, and RNAi-31 **(H)**. Abnormal pollen grains of RNAi-4 **(C)**, RNAi-14 **(F)**, and RNAi-31 **(I)** showed dim intine layers. Bar = 10 μm.

Supplementary Figure 7 Protein sequence analysis of mutated *AtNMDM1* alleles. **(A)** The genomic structure of the *AtNMDM1* gene from Arabidopsis. Exons are represented by black boxes. The length of the *AtNMDM1* gene is 1,344 bp. A conserved PC4-like region in the AtNMDM1 was predicted by the NCBI website. Purple boxes indicate PC4-like regions and their amino acid sequences are shown. **(B)** Protein sequences generated by mutated *AtNMDM1* alleles contain altered or truncated PRC (PC4-like region conserved) domains. Amino acids marked by red represent frameshift protein domains, and red dotted lines represent termination in advance. Yellow box indicates the sequence of PRC domains in the wild type.

Supplementary Figure 8 Characteristics of IP/MS data by using AtNMDM1-FLAG infused protein as a bait. **(A)** The identified protein length distribution. **(B)** The relationship between distinct peptide number and identified peptide number. **(C)** Venn diagram of the IP/MS data (Total IP protein genes) and three RNA-seq data sets, including total differential expression genes, upregulated genes, and downregulated genes. **(D–F)** GO enrichment analyses in three GO categories containing biological process **(D)**, cellular component **(E)**, and molecular function **(F)**. The IP/MS data were first enriched by the AgriGO tool. Subsequently, long GO lists were summarized by the REVIGO tool for reducing functional redundancies. Each bubble represents a non-redundant GO term. Using cartesian coordinates (x, y), shorter distance between bubbles indicates a closer relationship. Bubble size indicates the frequency of the GO term in non-redundant GO terms. Bubble color indicates the significance of enrichment (*P* < 0.01).
