# Supplementary figures and images for: Arabidopsis Novel Microgametophyte Defective Mutant 1 Is Required for Pollen Viability via Influencing Intine Development in Arabidopsis
Source: Front Plant Sci. 2022 Apr 12;13:814870. doi: 10.3389/fpls.2022.814870 (PMC9039731; doi:10.3389/fpls.2022.814870)

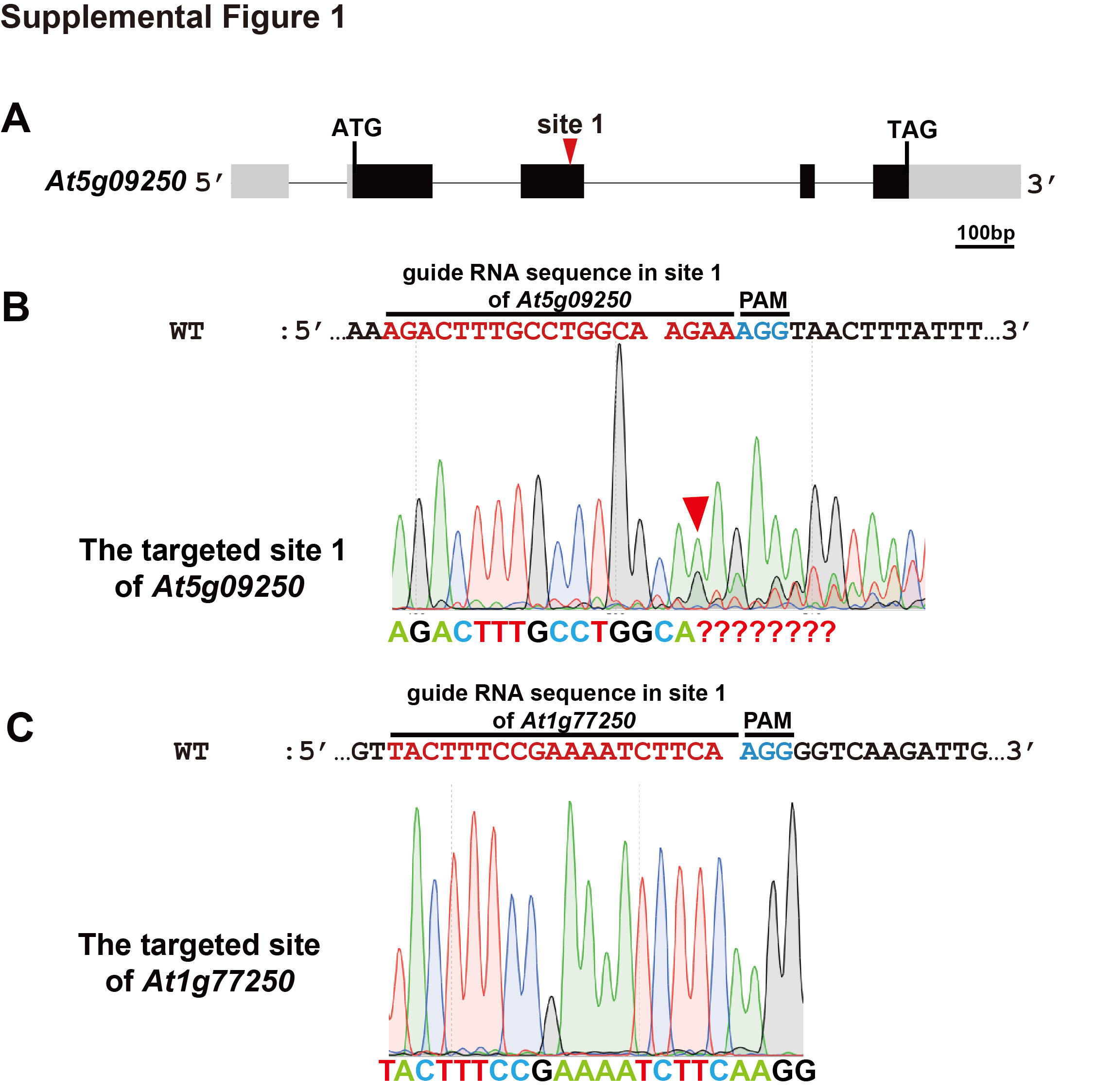

Supplement: Supplementary Figure 1 — Examples of direct sequencing PCR products containing targeted site 1 of AtNMDM1 in T1 transgenic plants. (A) The genomic structure of the AtNMDM1 from Arabidopsis. Exons are represented by black boxes. Introns are shown by a dashed line. The target site1 is indicated by a red arrow. The length of AtNMDM1 is 1344 bp. (B) The sequencing chromatograms with overlapping traces are indicated at the target site1 of At5g09250 by using the direct sequencing method in T1 individuals. The wild-type sequence is shown at the top with the PAM sequence highlighted in blue and the target sequence in red. (C) The target site1 of At1g77250 was not edited. [file Image_1.JPEG]

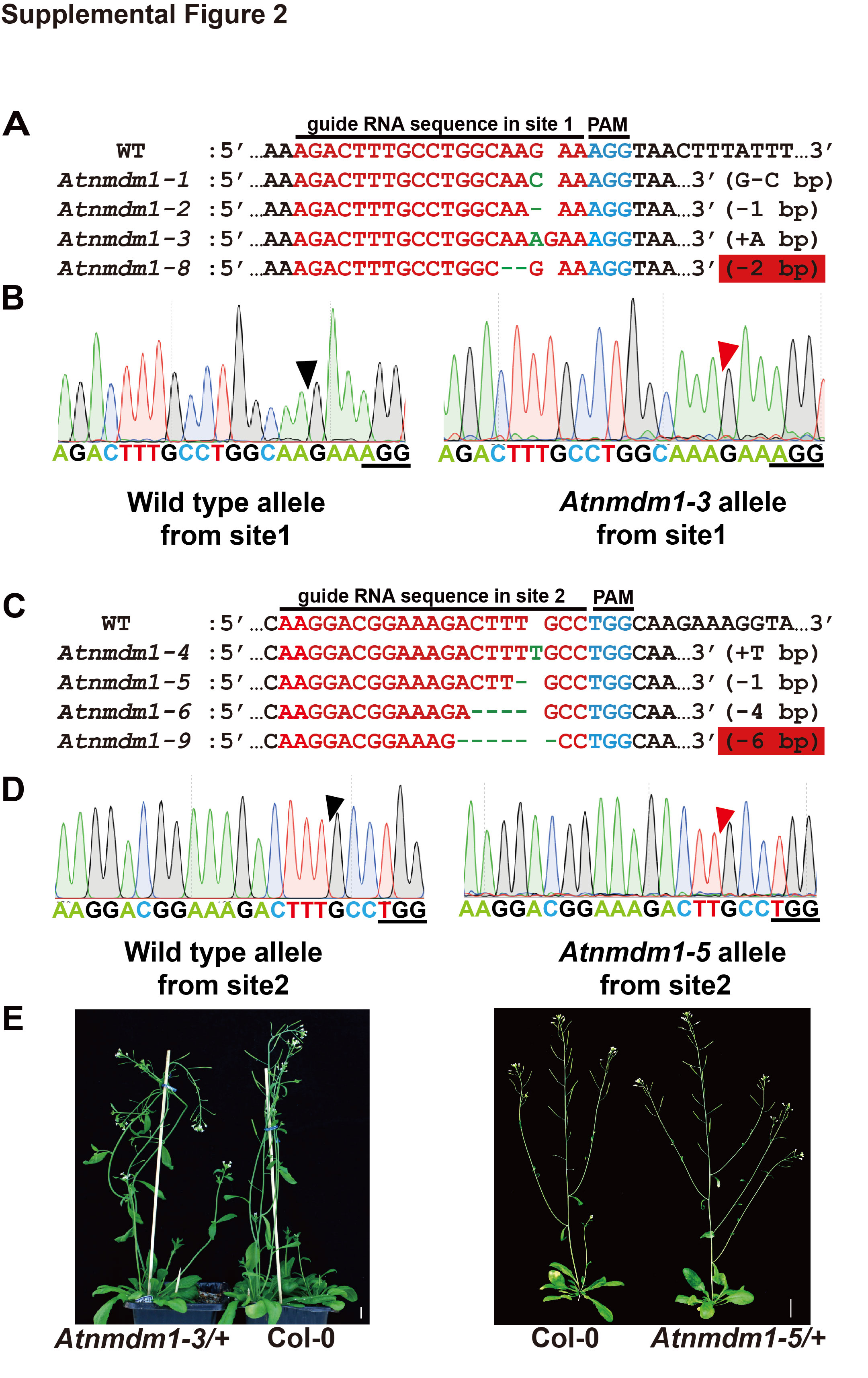

Supplement: Supplementary Figure 2 — New variants of AtNMDM1 were generated in T2 transgenic plants; and examples of sequencing chromatograms containing targeted sites, using a complex-sequencing method in T2 transgenic plants. (A,C) New mutations at AtNMDM1 were generated in T2 transgenic plants. The wild-type sequence is shown at the top with the PAM sequence highlighted in blue and the target sequence in red. Green dash and letter indicate deleted bases and insertions or changed bases, separately (+, insertion; −, deletion; letter-letter, changed); New variants are shown in red boxes. (B,D) Examples of sequencing chromatograms. Wild type allele and Atnmdm1-3 allele at the targeted site 1 (B) and wild type allele and Atnmdm1-5 allele at the targeted site 2 (D) are shown using a complex-sequencing method. Red arrows show the start positions where, or from where, the mutations occurred. PAM is shown by a black underline. Black arrows indicate the corresponding start positions from where the mutations occurred in the wild type. (E) Vegetative growth of wild type and AtNMDM1+ / − mutants, Atnmdm1-3/+, and Atnmdm1-5/+ were quite normal in the plants after sowing at 39 or 40 days. Bars = 2 cm (left), 1 cm (right). [file Image_2.JPEG]

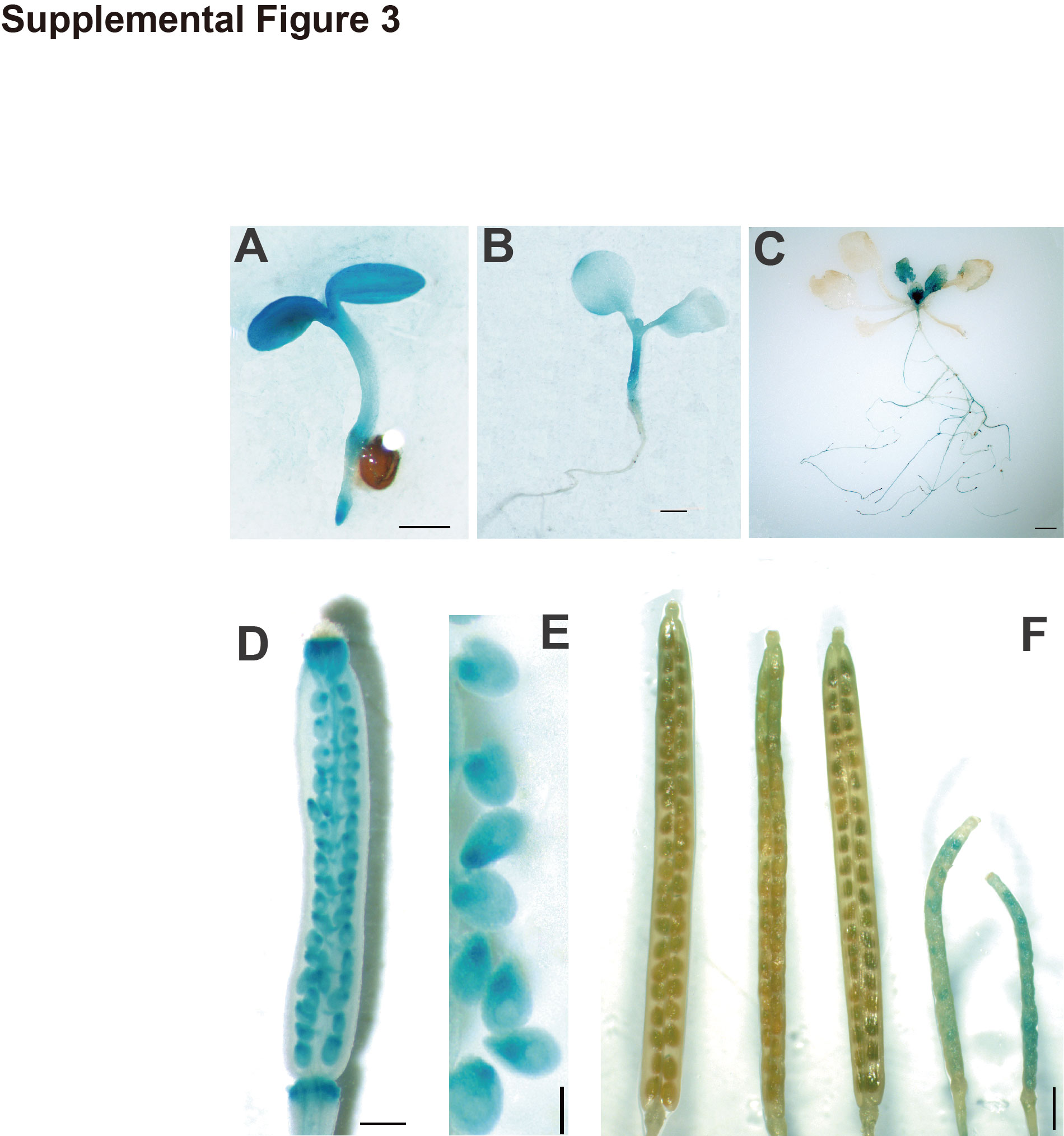

Supplement: Supplementary Figure 3 — AtNMDM1 promoters trigger GUS expression during the growth stage in Arabidopsis. (A–C) show 1–14 day ProAtNMDM1:GUS transgenic seedlings. (D) Young siliques after pollination. (E) Embryo in a young silique. (F) Different stages of siliques. GUS expression in transgenic plants carrying ProAtNMDM1: GUS is indicated in blue following staining for GUS activity. (A–D,F), bar = 1 mm; (E), bar = 500 μm. [file Image_3.JPEG]

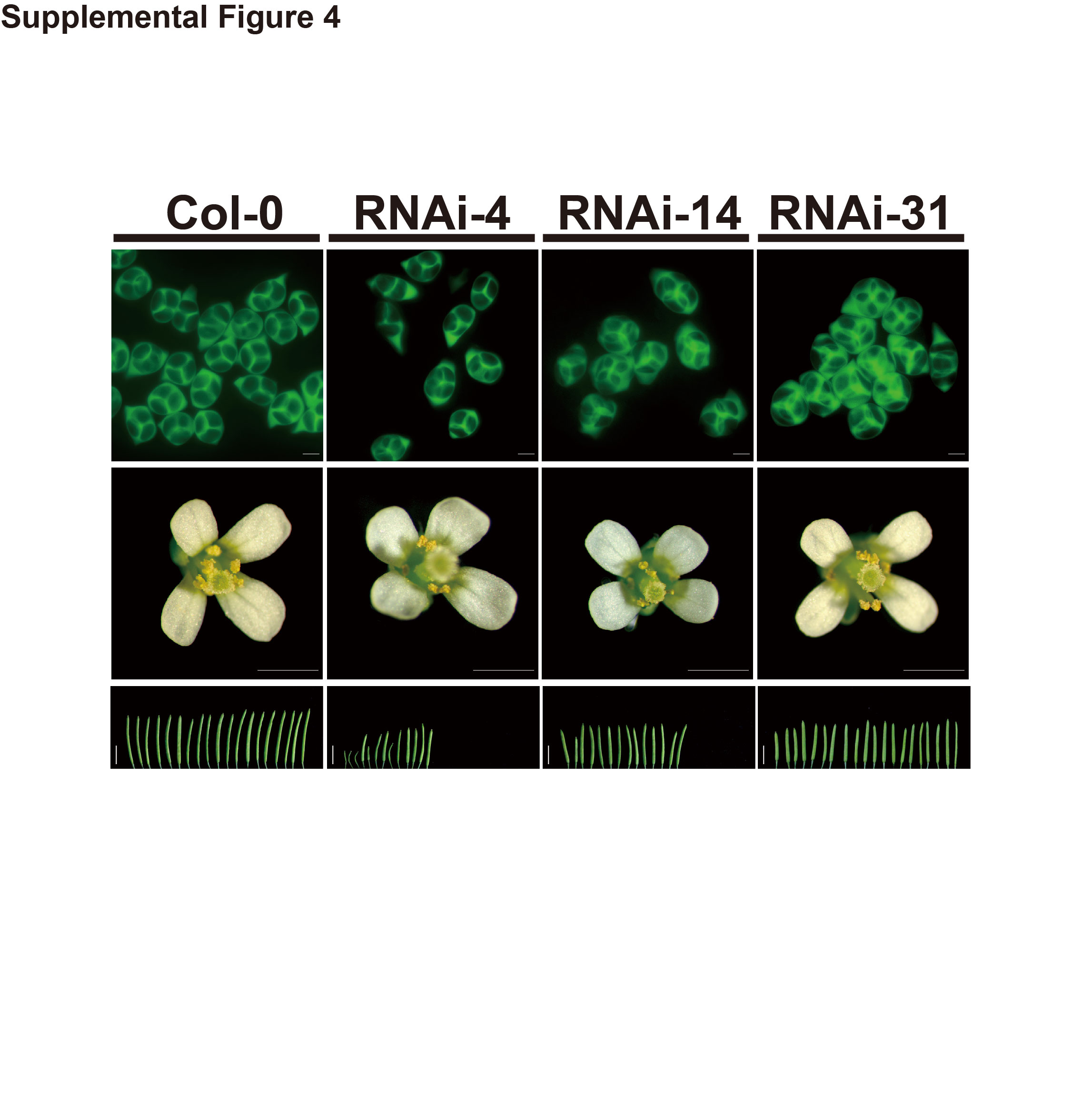

Supplement: Supplementary Figure 4 — Analysis of the phenotype of AtNMDM1 RNAi lines. From top to bottom, the callose wall in AtNMDM1 RNAi lines RNAi-4, RNAi-14, and RNAi-31 are quite normal around the microspores compared with the wild type ones (top); the number of pollen grains in AtNMDM1 RNAi lines was less than in the wild type (middle); the silique length of AtNMDM1 RNAi lines was obviously shorter than in the wild type (bottom). Callose wall, bars = 10 μm. Opened flower, bars = 1 mm. Silique, bars = 5 mm. [file Image_4.JPEG]

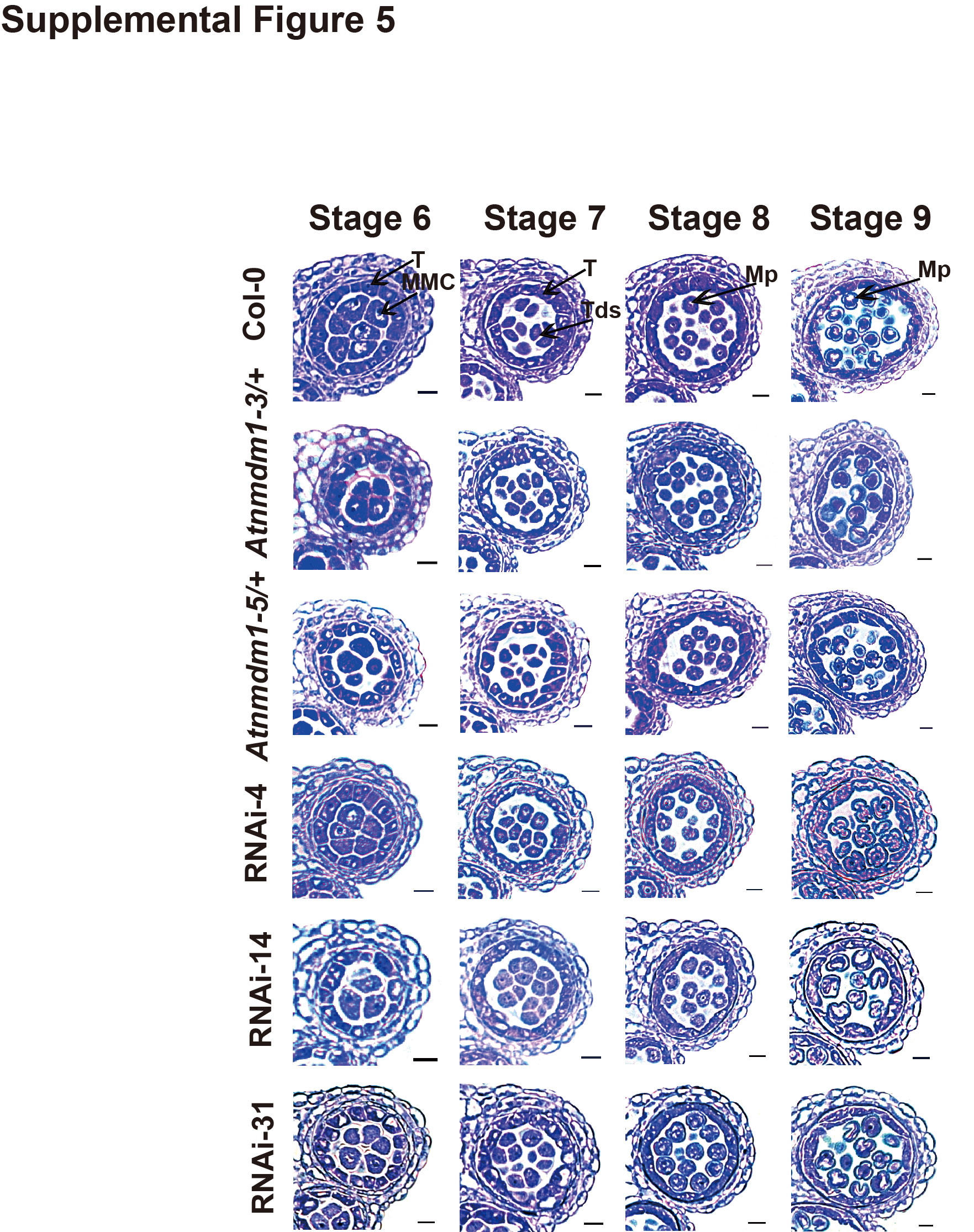

Supplement: Supplementary Figure 5 — The phenotype of AtNMDM1+ / − mutants and RNAi lines were normal-type in anther stages 6–9. Semi-thin cross-sections of anthers from wild type, Atnmdm1-3/+, Atnmdm1-5/+ and three AtNMDM1 RNAi lines RNAi-4, RNAi-14, and RNAi-31 were stained with toluidine blue. The microspores were quite similar for the wild type in Atnmdm1-3/+, Atnmdm1-5/+, and three AtNMDM1 RNAi lines—RNAi-4, RNAi-14, and RNAi-31—from stages 6 to 9. T, Tapetum; MMC, microspores mother cell; Tds, tetrads; Mp, microspore. Bars = 10 μm. [file Image_5.JPEG]

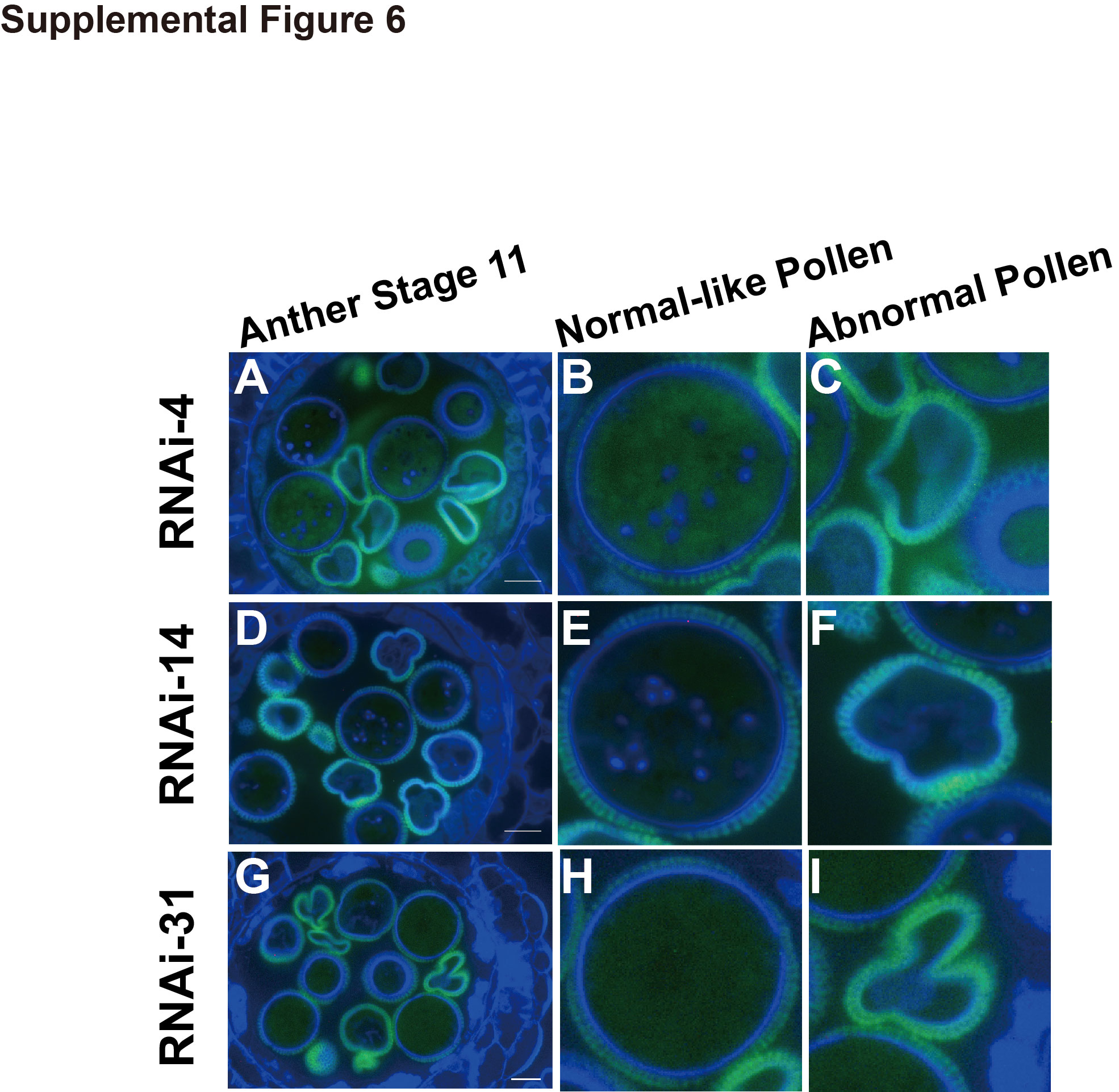

Supplement: Supplementary Figure 6 — Calcofluor white staining of semi-thin sections of AtNMDM1 RNAi lines. (A,D,G) present anther sections at stage 11 in the AtNMDM1 RNAi lines RNAi-4, RNAi-14, and RNAi-31, respectively. Calcofluor white staining revealed that all normal-like pollen showed a blue, fluorescent ring of the intine layer in the RNAi-4 (B), RNAi-14 (E), and RNAi-31 (H). Abnormal pollen grains of RNAi-4 (C), RNAi-14 (F), and RNAi-31 (I) showed dim intine layers. Bar = 10 μm. [file Image_6.JPEG]

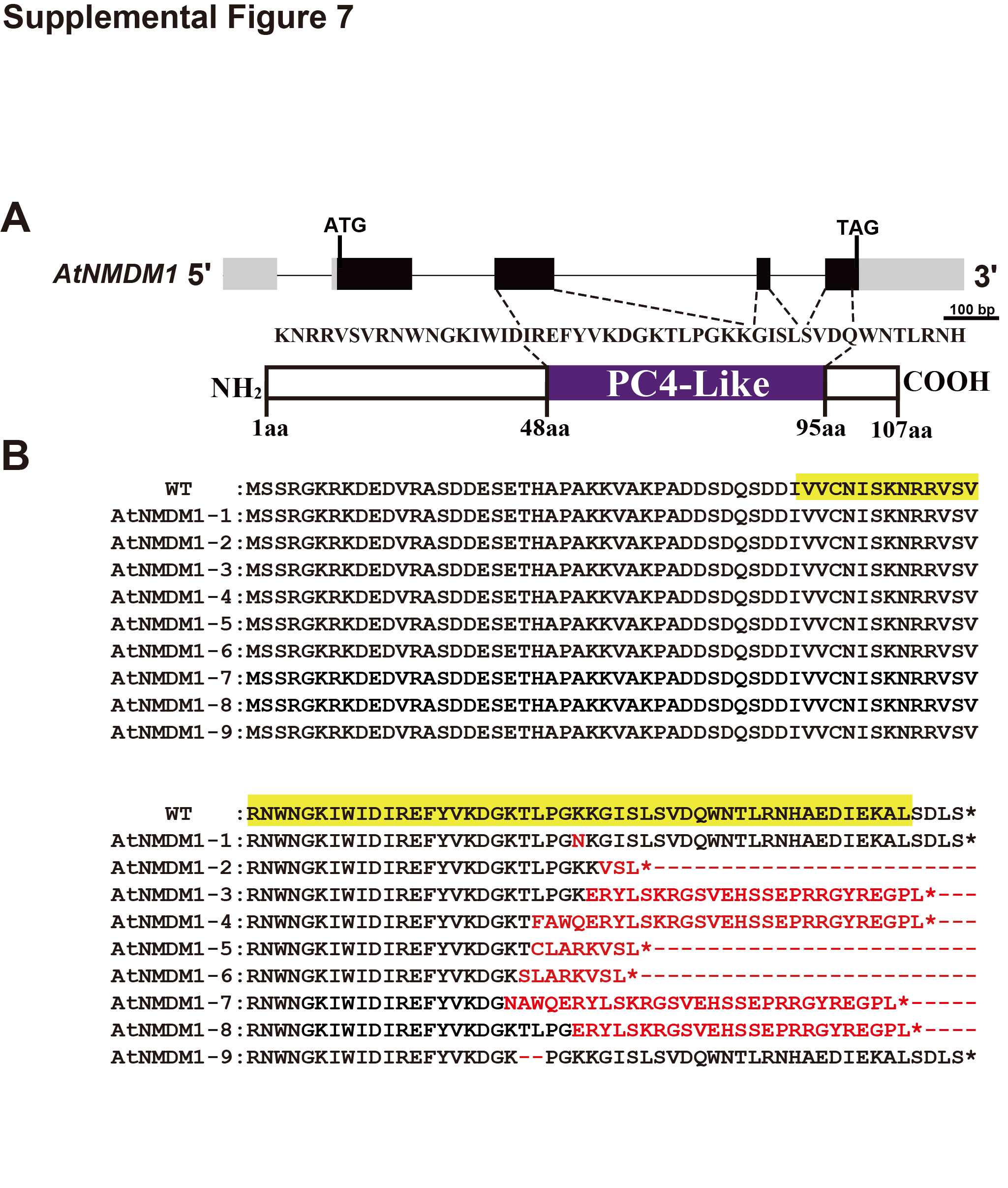

Supplement: Supplementary Figure 7 — Protein sequence analysis of mutated AtNMDM1 alleles. (A) The genomic structure of the AtNMDM1 gene from Arabidopsis. Exons are represented by black boxes. The length of the AtNMDM1 gene is 1,344 bp. A conserved PC4-like region in the AtNMDM1 was predicted by the NCBI website. Purple boxes indicate PC4-like regions and their amino acid sequences are shown. (B) Protein sequences generated by mutated AtNMDM1 alleles contain altered or truncated PRC (PC4-like region conserved) domains. Amino acids marked by red represent frameshift protein domains, and red dotted lines represent termination in advance. Yellow box indicates the sequence of PRC domains in the wild type. [file Image_7.JPEG]

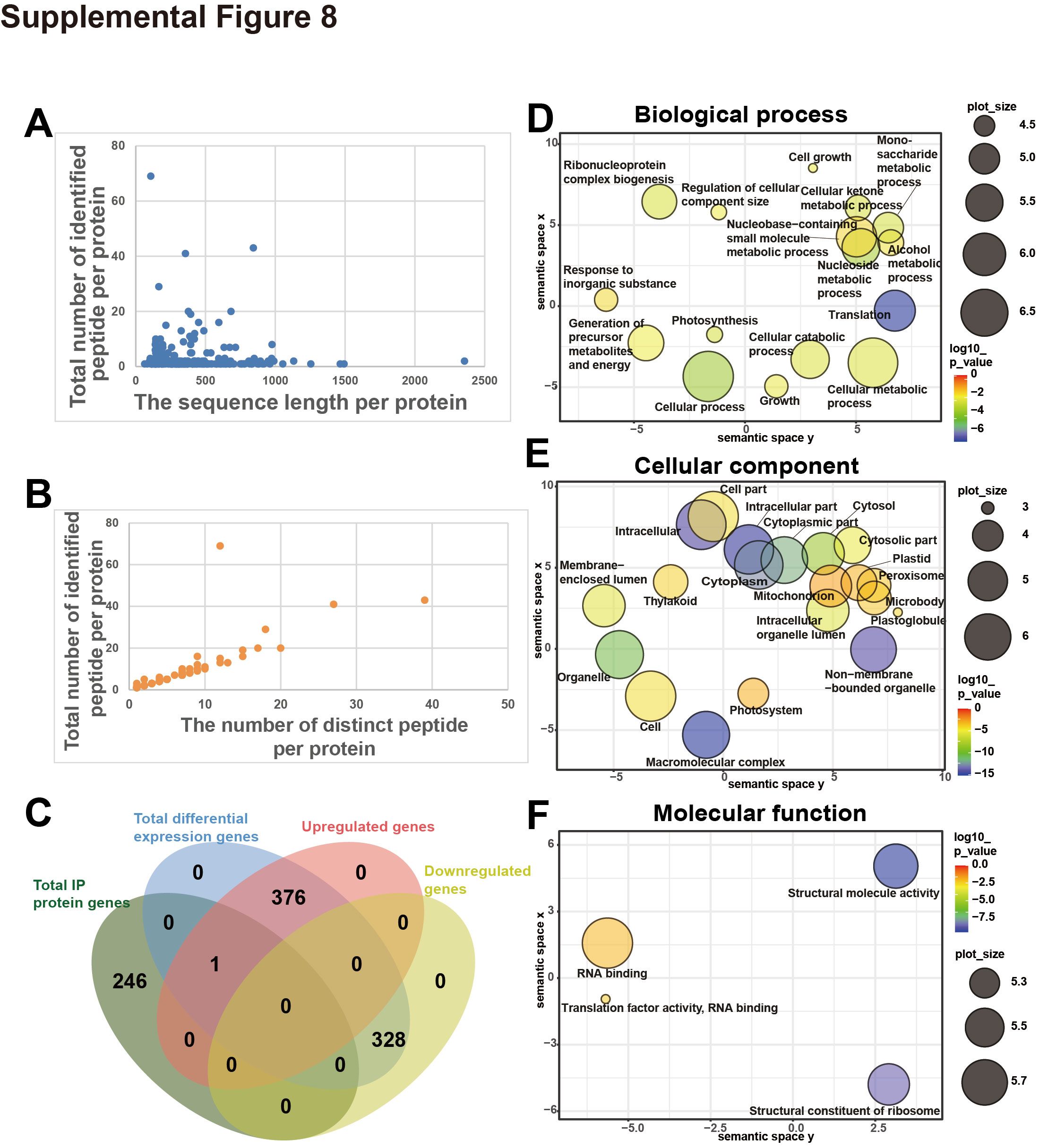

Supplement: Supplementary Figure 8 — Characteristics of IP/MS data by using AtNMDM1-FLAG infused protein as a bait. (A) The identified protein length distribution. (B) The relationship between distinct peptide number and identified peptide number. (C) Venn diagram of the IP/MS data (Total IP protein genes) and three RNA-seq data sets, including total differential expression genes, upregulated genes, and downregulated genes. (D–F) GO enrichment analyses in three GO categories containing biological process (D), cellular component (E), and molecular function (F). The IP/MS data were first enriched by the AgriGO tool. Subsequently, long GO lists were summarized by the REVIGO tool for reducing functional redundancies. Each bubble represents a non-redundant GO term. Using cartesian coordinates (x, y), shorter distance between bubbles indicates a closer relationship. Bubble size indicates the frequency of the GO term in non-redundant GO terms. Bubble color indicates the significance of enrichment (P < 0.01). [file Image_8.JPEG]
