# Supplementary material for: Arabidopsis Novel Microgametophyte Defective Mutant 1 Is Required for Pollen Viability via Influencing Intine Development in Arabidopsis
Source: Front Plant Sci. 2022 Apr 12;13:814870. doi: 10.3389/fpls.2022.814870 (PMC9039731; doi:10.3389/fpls.2022.814870)
Supplement: Supplementary file 9 [file Table_1.DOCX]

**Supplemental Table 1. Genetic transmission analysis of the *Atnmdm1-3* and *Atnmdm1-5* alleles.** Heterozygous mutants *Atnmdm1-3/+* and *Atnmdm1-5/+* plants (*AtNMDM1^+/-^*) were selfed or crossed with wild type plants (*AtNMDM1^+/+^* or *+/+*) as the female or male parent. Pollen from F2 plants was stained with Alexander stain. Aborted or non-aborted pollen grains of each plant were determined by microscopy. “n” represents the number of plants whose phenotype likes wild type or *AtNMDM1^+/-^* mutant.

| **Heterozygous mutant** | **Female × Male** | **Wild type (n)** | ***AtNMDM1^+/-^* (n)** | **% *AtNMDM1^+/-^*** |
| --- | --- | --- | --- | --- |
| *Atnmdm1-3/+* | *Atnmdm1-3/+* × *Atnmdm1-3/+* | 30 | 28 | 48 |
| *Atnmdm1-5/+* | *Atnmdm1-5/+* × *Atnmdm1-5/+* | 178 | 160 | 47 |
| *Atnmdm1-5/+* | *Atnmdm1-5/+* × *+/+* | 153 | 128 | 45 |
| *Atnmdm1-5/+* | *+/+* × *Atnmdm1-5/+* | 121 | 109 | 47 |
