# Supplementary material for: Arabidopsis Novel Microgametophyte Defective Mutant 1 Is Required for Pollen Viability via Influencing Intine Development in Arabidopsis
Source: Front Plant Sci. 2022 Apr 12;13:814870. doi: 10.3389/fpls.2022.814870 (PMC9039731; doi:10.3389/fpls.2022.814870)
Supplement: Supplementary file 10 [file Table_2.DOCX]

**Supplemental Table 2. The crosses analysis of *Atnmdm1-5/+* and wild type (WT).** The reciprocal crosses showed reduced female fertility in *Atnmdm1-5/+*. “n” represents the observed number of siliques examined for each hybrid combination. *, P-value <0.05; **, P-value <0.01; ***, P-value <0.001.

| **Female × Male** | **Seeds/per silique**  **(Mean±SD)** | **Observed number of silique (n)** | **P-value (compared with wild type)** |
| --- | --- | --- | --- |
| WT × WT | 54.98±4.07 | 306 | --- |
| *Atnmdm1-5/+* × *Atnmdm1-5/+* | 26.09±3.97 | 637 | *** |
| WT × *Atnmdm1-5/+* | 45.61±4.09 | 87 | *** |
| *Atnmdm1-5/+* × WT | 25.71±4.22 | 203 | *** |
